# An Assembly Funnel Makes Biomolecular Complex Assembly Efficient

**DOI:** 10.1371/journal.pone.0111233

**Published:** 2014-10-31

**Authors:** John Zenk, Rebecca Schulman

**Affiliations:** 1 Chemical and Biomolecular Engineering, Johns Hopkins University, Baltimore, Maryland, United States of America; 2 Computer Science, Johns Hopkins University, Baltimore, Maryland, United States of America; Weizmann Institute of Science, Israel

## Abstract

Like protein folding and crystallization, the self-assembly of complexes is a fundamental form of biomolecular organization. While the number of methods for creating synthetic complexes is growing rapidly, most require empirical tuning of assembly conditions and/or produce low yields. We use coarse-grained simulations of the assembly kinetics of complexes to identify generic limitations on yields that arise because of the many simultaneous interactions allowed between the components and intermediates of a complex. Efficient assembly occurs when nucleation is fast and growth pathways are few, *i.e.* when there is an assembly “funnel”. For typical complexes, an assembly funnel occurs in a narrow window of conditions whose location is highly complex specific. However, by redesigning the components this window can be drastically broadened, so that complexes can form quickly across many conditions. The generality of this approach suggests assembly funnel design as a foundational strategy for robust biomolecular complex synthesis.

## Introduction

Within cells, bottom-up phenomena organize biomolecules into structures with sizes ranging from angstroms to microns. Precise control over structure at the angstrom and nanometer scales is important for optimizing catalysis [1], [2], the action of molecular machines [3] or molecular recognition [4]. Larger biomolecular structures orchestrate processes such as translation, adhesion, or controlled transport. One goal of chemistry and molecular engineering is therefore to develop analogous bottom-up methods for controlling biomolecular structure across the same range of dimensions [5], [6].

Different physical processes are responsible for the *in vivo* formation of structure across these length scales. Stable nanometer- or angstrom-scale structures generally form as the result of folding a protein or RNA chain with a particular sequence [2]. Folding larger structures from a single chain is difficult because synthesizing long, sequence-specific polymers without errors is a challenge [7] and the potential for a folding process to become frustrated increases quickly with polymer length [8], [9]. Larger structures instead form through a hierarchical assembly process in which folded components self-assemble together into a larger complex. Examples of such complexes include the ribosome, proteasome and antibodies. Some complexes, including the nuclear pore complex [10], cell adhesions [11] or the kinetochore [12] can contain hundreds of components and reach sizes of more than a micron. Complex formation is ubiquitous: in *Escherichia coli*, for example, more than 20% of known polypeptides become reported members of protein complexes [13].

While the development of strategies for the design of synthetic self-assembling complexes have long lagged behind the design of folding processes, recently, a wealth of designed complexes assembled from proteins [14], nucleic acids [15] and other components [16], [17] has spurred interest in developing rules and general strategies for designing complexes [18]–[20]. Generally, design methods attempt to maximize complex yield by maximizing the thermodynamic stability of the complex or the free energy difference between the complex and other potential structures with the inherent assumption that thermodynamic equilibrium will be achieved [20]–[23]. Yet, in practice, complexes that are thermodynamically stable often assemble with low yields or may take as long as weeks to assemble properly [24]–[26]. While unaccounted-for experimental effects such as stoichiometric imbalances between components [27] might explain lower yields or slower than expected assembly times, kinetic factors that could limit yield are rarely investigated. To improve yields and dynamics, there are currently few strategies other than complex redesign [28] when thermodynamic design considerations fail.

Here, we test the assumption that self-assembly processes for biomolecular complexes generally reach a high-yield equilibrium state by simulating the kinetics of a variety of generic, idealized assembly reactions. We find that for typical biomolecular complex self-assembly reaction rates and component concentrations [29]–[32], it may take days or weeks to reach a state close to equilibrium, even when equilibrium yields are low. Thus, design processes that rely solely on thermodynamics to predict yields may meet with mixed success because yield is limited kinetically rather than thermodynamically. Our simulations also identify two key reasons why some self-assembly processes can be slow. First, near the melting temperature of the complex, low nucleation rates limit the rate of formation of complexes. Second, far below the melting temperature, assembly may occur rapidly through many different pathways, combinatorially trapping intermediate assembly products. Once assembly reaches this trapped state, complexes can form only after intermediates disassemble, which can be very slow. Avoiding both of these regimes is required to achieve high yield. For many common complexes, this requirement means that complex formation happens efficiently only under a narrow range of physical conditions. We show that designing components that skirt such kinetic pitfalls can significantly speed up assembly and enhance yields.

## Results

### A simple model of multicomponent biomolecular complex self-assembly

**Figure 1:**
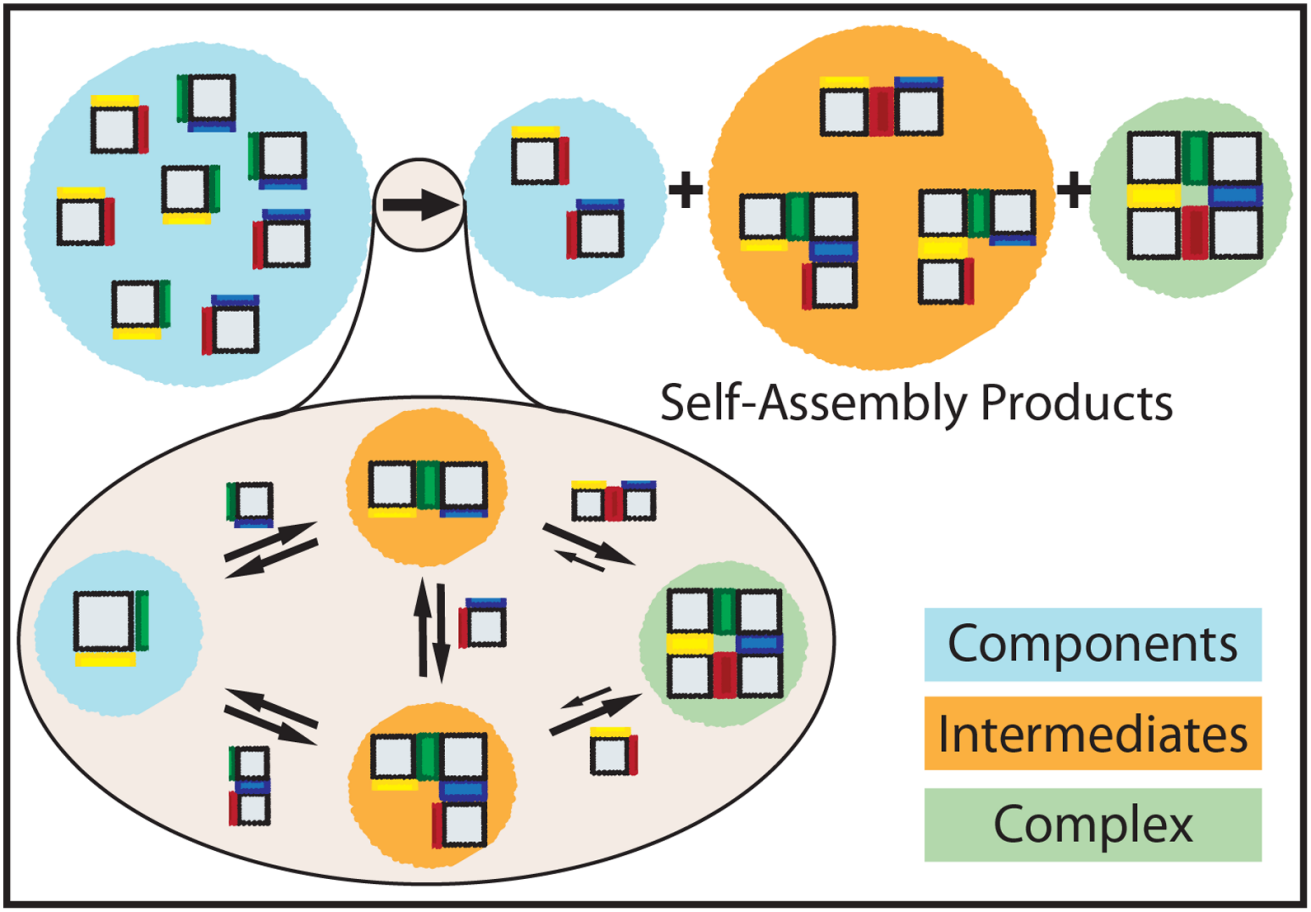
Self-assembly model for a 4-component square grid (“2×2”) complex. The square, rigid components have specific binding rules on each edge denoted by edge colors. Like colored edges interact, whereas edges with different colors and black edges do not interact. An initial, fixed number of components is depleted during self-assembly. At the end of the process, the solution contains a mixture of components, intermediates and complexes.

To characterize the kinetics of complex assembly, we use a simple model of assembly in which rigid components of a generic complex bind to one another via orientation-specific pairs of complementary interfaces (Fig. 1). We assume that all components have identical interaction energy at each interface and the same initial concentration. Interaction between non-matching interfaces, or crosstalk, is neglected, reflecting rapid advances in the design of specific biomolecular interfaces [14], [24]–[26], [33], [34]. Multiple different rigid components and their unique interfaces could be easily fabricated from DNA, for example, using existing techniques such as DNA origami [27] or DNA bricks [25]. In our model, we consider all binary reactions that produce a complex or any connected subset of components, which we call intermediates (see Supporting Methods).

Our simulations use on and off rates similar to those measured for oligonucleotide [32], protein [30], DNA tile [31], and ribosomal subunit-RNA [29] reactions. Simulated assembly protocols are simple and modeled after those in broad experimental use [26]–[28]. Assembly timescales are realistic, ranging from 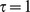, or about 30 minutes to 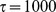, or about 2.7 weeks for 1 nM of components (*i.e.,* concentrations typical for large (megadalton) DNA nanostructures [27]), or 30 minutes for 1 uM of components (see Equation 1). To model the interplay of changes in bond energy that could result from multi-bond reactions (*e.g.,* from entropic or allosteric effects [35]), we introduce a dimensionless bond coupling term *α* that determines how the free energy of interaction scales with the number of bonds formed (see Equation 2). We use the dimensionless parameter 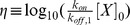 as an analog for inverse temperature (*e.g.,* high values of 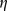 correspond to low temperatures and strong interactions and vice versa) and define yield as the fraction of total material in complete complexes (see Equation 3).

The goal of our study is to understand how yields of self-assembled biomolecular complexes vary with complex size (in terms of number of components), geometry and reaction parameters (*e.g.*, 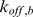, 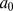) by using kinetic simulations and as a result, learn how to design complexes and assembly protocols to increase yields. In order to elucidate general principles, we focus on a set of generic complexes: 1-dimensional “line” complexes of different lengths, 2-dimensional square “grid” complexes with different numbers of components on a side and a 3-dimensional “cube” complex.

**Figure 2:**
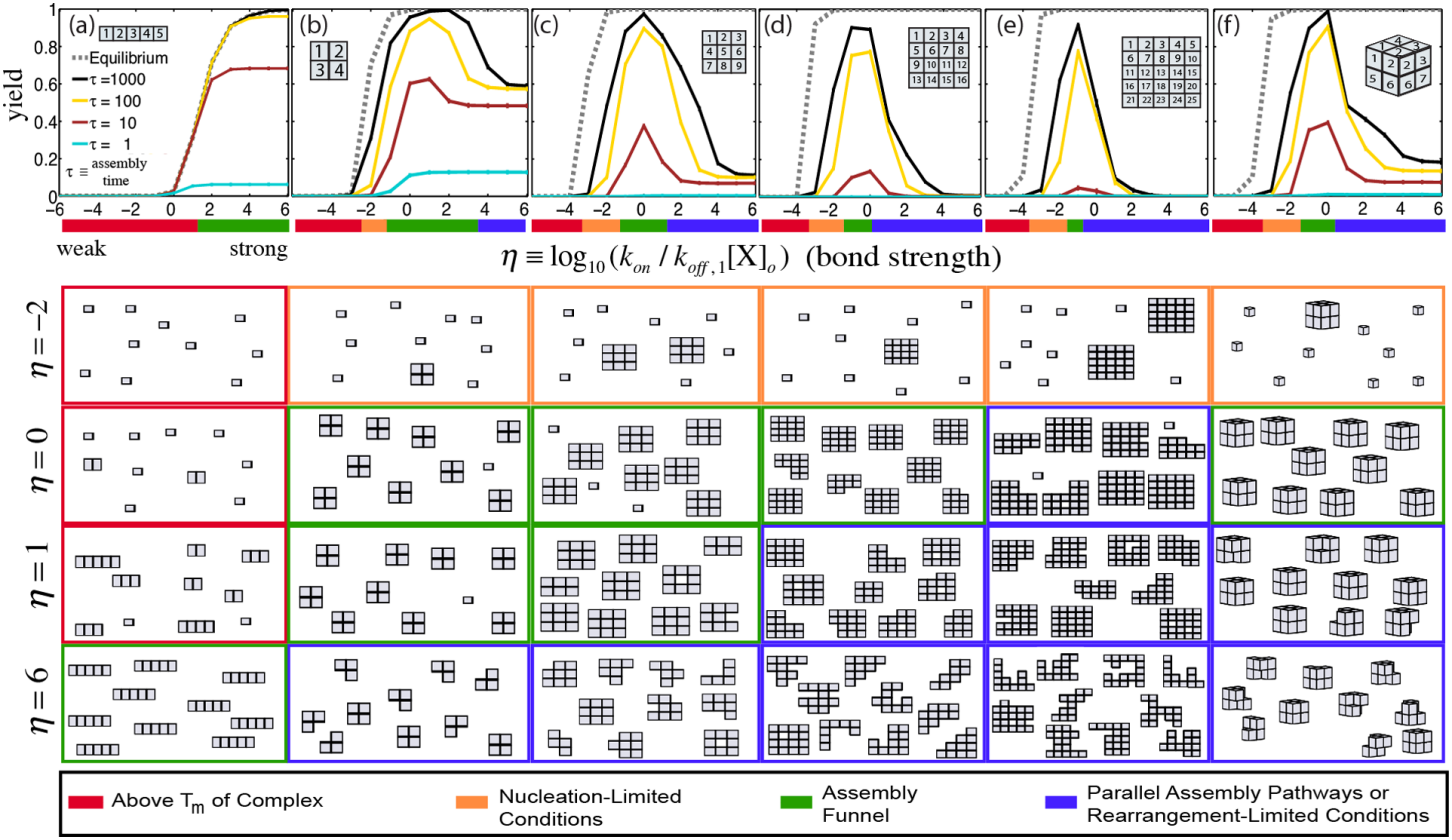
Thermodynamic equilibrium is a good predictor of yield for isothermal assembly after long assembly times for 1-dimensional complexes, but not 2- or 3-dimensional complexes. Assembly yields for a (a) 1×5 line complex, (b) 2×2, (c) 3×3, (d) 4×4 and (e) 5×5 square grid complex and (f) 2×2x2 cube complex as a function of the dimensionless temperature parameter, 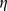. Inset diagram depicts the complex. Numbers on the components in the complex indicate component identity (*e.g*. component “1” is different than component “2”). The dashed line indicates thermodynamic equilibrium. Dimensionless reaction time is defined as 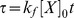 where 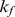 is the macroscopic forward reaction rate constant and 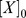 is the initial concentration of components. Colored bars and boxes below figures represent the four different assembly regimes (Text S3). The assembly funnel regime is considered to be where the complex is thermodynamically favored (*i.e.,* 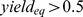) and assembly is rapid such that 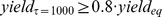. Assembly “snapshots” (below graphs) are taken at 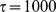 and 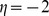 (top row), 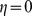, 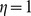, and 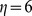 (bottom row) and comprised of ten random species drawn from the reaction mixture, weighted by concentration (Text S4). Error bars indicate the standard deviation of the reported quantity after 10 simulations and where omitted, are <1%. Here and elsewhere unless otherwise noted, there is no bond coupling (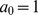).

Estimating the yield of a complex by considering its free energy relative to the free energies of other potential products is a standard method of estimating the yield of a self-assembly reaction [36], but such estimates are relevant only when assembly reactions are close to equilibrium. To determine whether typical reactions approach equilibrium, we modeled the kinetics of assembly using component concentrations, reaction times and rates typical of experimental self-assembly reactions [29]–[32]. To understand the effects of temperature, we initially studied reactions that take place at a single temperature (a single value of 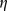). Isothermal assembly of 1D line complexes quickly achieved yields near those predicted at thermodynamic equilibrium for all interaction energies considered (Fig. 2a and Figs. S3 and S4). The system as a whole also approached equilibrium, as demonstrated by the concentrations of both complexes and intermediates (Fig. S5). Yields of line complexes were highest when the interactions between components were strongest, in agreement with both thermodynamic predictions and similar studies of self-assembly kinetics [37].

### Strong interactions maximize yield for 1-dimensional *systems only*

While strong interactions maximize the yield of line complexes, strong interactions in even small grid or cube complexes with no bond coupling (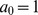) produced yields far lower than yields expected at equilibrium for simulated reaction times as long as weeks (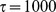, ∼2.7 weeks for 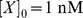) (see Figs. 2b-f and Figs. S8 and S9). Further, after a certain point, increasing reaction time only marginally increases yield (Figs. S11, S22). For example, increasing the assembly time from 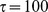 to 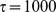 increased the yield of 3×3 grid complexes by at most ∼10%. Similarly marginal increases in yield were observed when assembly times were increased further to 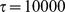 (Fig. S10). These results suggest that these self-assembly processes rarely approach the equilibrium state in practice.

### Slow nucleation and molecular rearrangement rates can limit yield

To understand why grid and cube complexes assembled so slowly, we investigated the composition of the simulated solution after the completion of the reaction (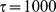) under many isothermal assembly conditions (Fig. 2, and Figs. S13, S15 and S23). Above the melting temperature of a given complex, no complexes form. Just below the melting temperature, the most abundant species aside from complexes were components, suggesting that yield under these highly reversible conditions is limited by the long times required to nucleate intermediates. Under effectively irreversible conditions (*i.e.*, high values of 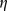), intermediates that cannot interact with one another to form complexes were the most common species, including the four 3-component intermediates in the 2×2 square grid complex and the 5 to 8-component intermediates in the 3×3 square grid complex (Fig. S14). Under these conditions, components or smaller intermediates must detach from a larger intermediate and attach to another intermediate, or “rearrange”, in order to complete a complex, which is an energetically unfavorable and therefore slow process. This rearrangement-limited regime is present for the assembly of grid and cube complexes but not line complexes because the intermediates to line complexes never need to rearrange to produce complexes. These results are corroborated by studies of viral capsid assembly [38] as well as homomeric [39]and ring-like protein complex assembly [40], where nucleation and rearrangement rates were found to influence assembly efficiency and fidelity.

### A high-yield assembly funnel regime occurs at medium-strength component interactions

The results thus far indicate that the self-assembly of grid and cube complexes could occur with high yields when bond strengths are neither too weak for fast nucleation nor too strong to prevent components in intermediates from rearranging. Indeed, our simulations show that there is a small window of medium component-component interaction strength where complexes are stable and assemble with high yields without requiring infeasibly long assembly times. We called this regime the “assembly funnel” regime, because in this regime the energy landscape contains a small number of smooth downward paths to complete complex formation, similar to a protein folding funnel [41] or a protein binding funnel [42]. This regime for grid and cube complexes is generally near 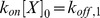 or 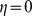. In our simulations of 2×2 to 5×5 square grid complexes, we found that increasing complex size shrinks the size of the already small assembly funnel regime by disfavoring forward conditions (*i.e.,* where 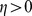). Increasing complex size increases the number of ways components can become “stuck” in incompatible intermediates, so completing a larger complex requires more molecular rearrangement on average than completing a smaller one.

### Reaction conditions determine the set of possible assembly pathways

**Figure 3:**
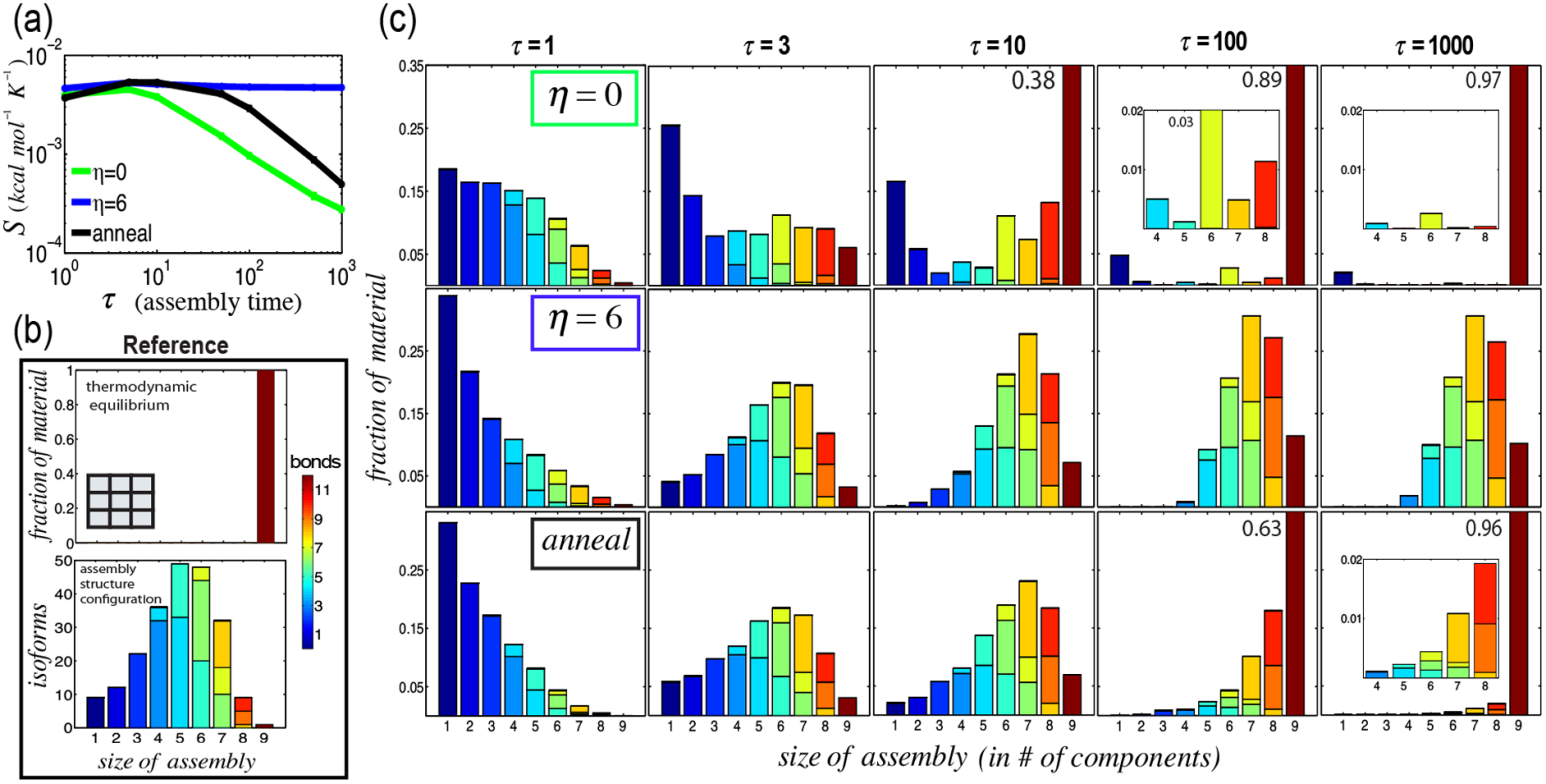
An assembly funnel means that complex assembly occurs via a small number of pathways. The possible set of reaction pathways govern assembly outcome under rearrangement-limited conditions, whereas thermodynamically favorable pathways govern assembly outcome in the assembly funnel regime. (a) Conformational entropy (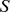) of the system under different assembly conditions as a function of assembly time, 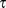. (b) Reference energy distributions of a 3×3 square grid complex based on thermodynamics and assembly configuration. Color spectrum indicates the number of bonds in an assembly. (c) Partition of energies at different times during self-assembly in the assembly funnel regime at 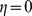 (green box), rearrangement-limited conditions at 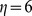 (blue box), and during an anneal (black box). Over the course of an anneal, 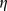 transitions from -6 to 6, spending 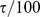 at 100 different linearly decreasing isothermal conditions. Values at the top right are complex yields. Inset plots show detail. Error bars <1%.

To further understand the influence of pathways on complex formation, we examined the kinds of intermediates that tend to arise and persist by measuring the conformational entropy, or distribution of species sizes and free energies, of the system. The conformational entropy is given by 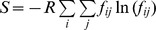 where 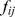 is the fraction of species with energy 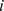 and 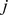 components. Higher values of conformational entropy correspond to broader distributions of assembly sizes and free energies. Under rearrangement-limited conditions, conformational entropy initially increases as many different intermediates form, and then plateaus (Fig. 3a). The species that form and remain are those that are most easily accessible via reaction pathways rather than those that are energetically favorable (Fig. 3b, c). In contrast, assembly in the assembly funnel regime favors the production of a relatively small number of intermediates, those lowest in free energy, so conformational entropy decreases with time as these low-energy intermediates and complexes form. Because complex size and geometry determine the possible reaction pathways and the types of assembly intermediates that can form [43], they also control the propensity of an assembly process to become “stuck” under a given set of reaction conditions.

### The time spent in the assembly funnel regime determines the yield

**Figure 4:**
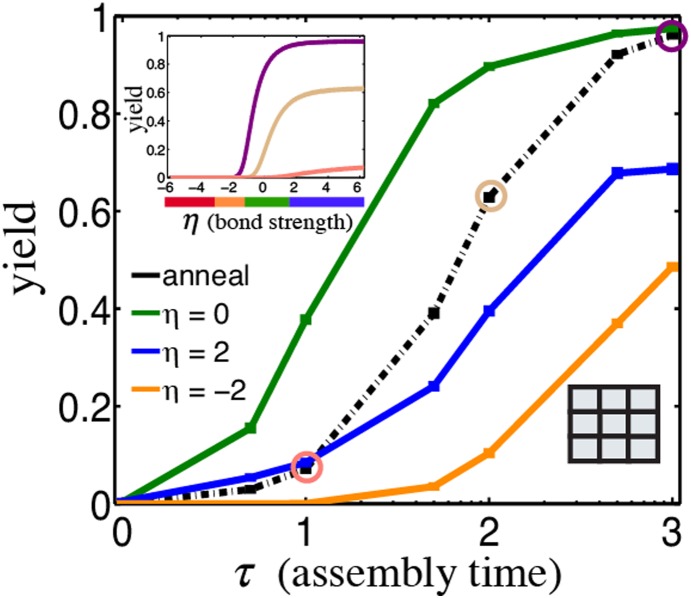
Complexes form rapidly in the assembly funnel regime. Yield of 3×3 square grid complex as a function of reaction time by assembling via annealing and at various isothermal assembly conditions: 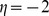 (orange, nucleation-limited), 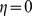 (green, assembly funnel) 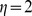 (blue, parallel assembly pathways and rearrangement-limited). Inset plot (top left) depicts yield during an anneal as a function of interaction strength for different reaction times: 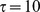 (salmon), 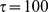 (beige), and 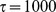 (purple). Inset diagram (bottom right) depicts the complex.

While complexes form quickly in the assembly funnel regime, the specific reaction conditions that generate an assembly funnel depend on the set of possible reaction pathways as well as kinetic and thermodynamic parameters that are generally unknown and difficult to estimate. One solution to this problem is to assemble via annealing. A typical annealing protocol begins at a temperature above the melting temperature of the complex, which is then gradually decreased until effectively irreversible conditions are achieved. To determine how yields using this protocol compare to those during isothermal assembly, we simulated annealing for square grid complexes. We found that yields during an anneal are predominately determined by the amount of the time spent in the assembly funnel regime. As the temperature decreases, few complexes form before the assembly funnel regime is reached. Within the funnel regime, complexes form rapidly, primarily through thermodynamic pathways (Figs. 3, 4 and Figs. S16–S18). After the annealing moves out of the assembly funnel regime, complexes are stabilized, but relatively few new complexes form. Thus, assembly via annealing is relatively efficient even when it is not known which conditions that generate an assembly funnel, which is in agreement to recent computational findings on DNA brick self-assembly [44]. However, to produce high yields, an anneal must be slower than a comparable isothermal assembly process in the assembly funnel regime because complex formation is slow for the majority of the anneal. This effect becomes more pronounced as complex size increases because the range of reaction conditions that produce an assembly funnel decreases. Thus, for very large complexes, it may be important to find ideal isothermal conditions, even when annealing is a practical option for assembly [28].

### Just a small amount of bond coupling between components is needed for high yield

**Figure 5:**
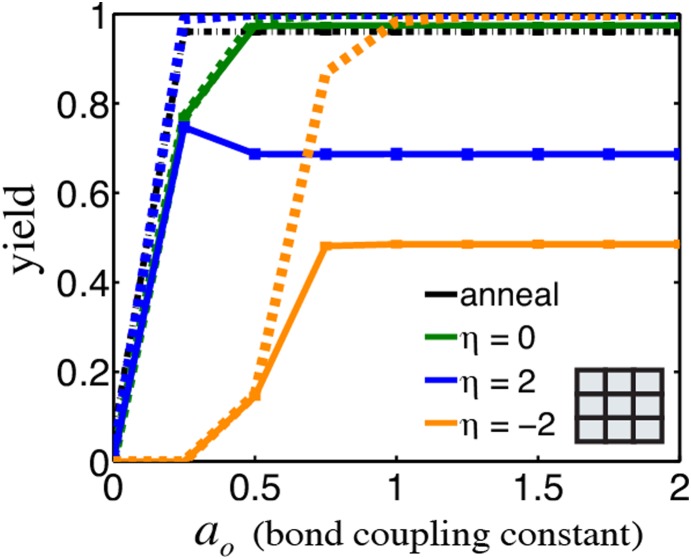
The amount of bond coupling, or additivity of bond energies during cooperative binding steps does not significantly affect assembly yields above a small threshold. Yield of a 3×3 square grid complex as a function of the bond coupling constant, 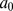 under many isothermal assembly conditions (solid lines, color) and after an anneal (black) for reaction time 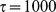. Dashed lines show yields at thermodynamic equilibrium for isothermal conditions with the same color. Error bars <1%.

2- and 3-dimensional complexes are generally stabilized by the interactions of multiple bonds between components, and the specific free energy changes that result from multi-bond interactions also shape the energy landscape for assembly [45]. To determine how the free energy of multi-bond interactions influences yield, we characterized changes in yield as we altered the coupling between multiple interfaces on a component. Surprisingly, we found that bond coupling was not an important determinant of assembly yield (see Fig. 5 and Figs. S7, S21). Although positive coupling (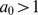) slightly broadens the set of conditions where complex yields are high at thermodynamic equilibrium (Figs. S6, S20), it leads neither to increased nucleation rates nor component rearrangement rates and thus does not increase yields in practice. Negative coupling (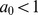) does not always reduce yields in the assembly funnel regime and can even marginally enhance yields under rearrangement-limited isothermal conditions by destabilizing some intermediates (Text S5). Thus, high-yield assembly can be obtained under the proper assembly conditions for a wide range of bond coupling values, as any coupling value is subject to equal pressures on nucleation and rearrangement rates.

### Components can be designed to assemble efficiently because they assemble *via* an assembly funnel under most conditions

**Figure 6:**
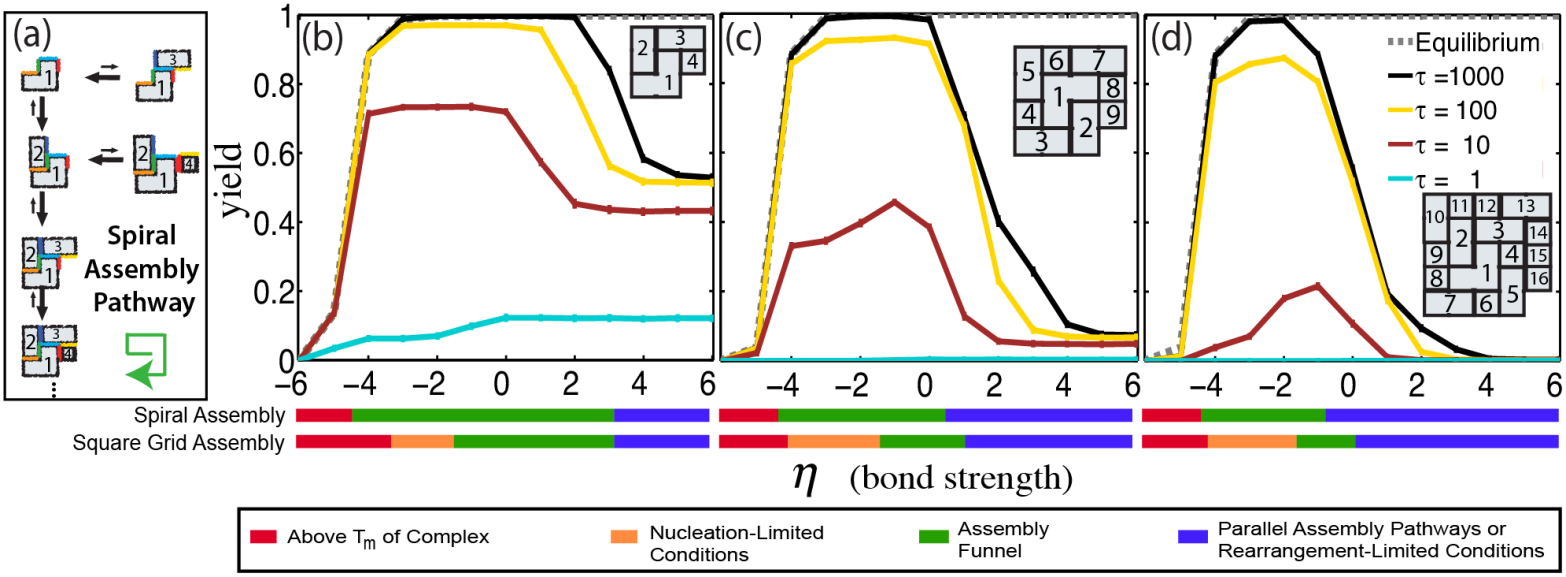
Design of components so that particular assembly pathways are favored can drastically increase assembly yields. (a) Schematic of spiral complex assembly via the favored assembly pathway. On the favored assembly pathway, assembly begins with the “L” shaped component, labeled “1”. At each assembly step, a component attaches through two interfaces (following the green arrow). Other components can only attach through one. Lengths of reaction arrows indicate propensities in the assembly funnel regime. Assembly yields for a (b) 2×2 (4 component), (c) 3×3 (9 component) and (d) 4×4 (16 component) spiral complex as a function of a dimensionless temperature parameter, 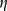. Inset diagram depicts the complex and numbers on the components in the complex indicate component identity. Colored bars below the figure represent the four different assembly regimes for spiral complexes and grid complexes containing the same number of components. Error bars <1%.

While it is challenging to optimize reaction conditions to produce high yields, might it be possible to create components that broaden the assembly funnel regime and thus self-assemble a desired complex more efficiently? To address this question, we designed components for a 2-dimensional target structure that were expected to have a smaller barrier to nucleation than the components of the grid complex we studied above. In a “spiral complex,” a spiral-shaped growth pathway allows all components to attach to the growing assembly via multiple bonds, so that there is no nucleation barrier to assembly. Because all other growth pathways require that components interact with one another via a single bond, the single spiral-shaped growth pathway is favored (Fig. 6a). Compared to square grid complex counterparts, the 4-, 9- and 16-component spiral complexes assemble faster and even achieve thermodynamic equilibrium in nucleation-limited regimes, broadening the reaction conditions that generate an assembly funnel (Fig. 6b–d). As a result, an anneal produces complexes more quickly, by almost an order of magnitude (Figs. S11 and S12). While the spiral scheme does not improve yield in the rearrangement-limited regime, this exercise suggests that effective self-assembly design strategies will likely promote rapid, high-yield complex formation by considering reaction pathways as well as nucleation and rearrangement rates.

## Discussion

Most existing strategies for the design and analysis of self-assembly processes use the thermodynamics of a complex as a starting point for predicting structure and yield. This strategy has been successful for understanding the assembly process of homogeneous or periodic crystals and superlattices [46]. While in principle, these strategies can be extended to guide the design of finite, heterogeneous complexes, we find that for a large class of multicomponent assembly processes, these strategies are insufficient because assembly is kinetically limited. Our results are echoed by experimental studies in which complex yields are low even when the desired product is strongly thermodynamically favored [25], [27], and in which assembly can be made efficient by assembling at a constant temperature at which assembly is optimal [28], in what we term the assembly funnel regime, if such a regime can be found. In fact, the assembly funnel assembly strategy has been used in the self-assembly homogenous multicompartment micelles [47].

Although optimizing assembly conditions appears difficult, this work suggests that it may be much more productive to design components such that they assemble efficiently through one or a small number of reaction pathways. This strategy of designing components that assemble efficiently appears to be important *in vivo*, as the components of protein complexes are under evolutionary pressure to assemble via ordered pathways [48].

One major assumption in this work is perfectly formed components: we do not address the challenge to form the components in the first place. In successfully forming biomolecular complexes, components must first be properly synthesized and folded or fabricated before they can associate to form a complex. Components that misfold or degrade can alter the assembly landscape by allowing the possibility of nonspecific interactions (*e.g.*, resulting in aggregated products, as clearly evidenced by diseases such as amyloidosis), which provides another, perhaps even larger, challenge in understanding complex assembly.

While this work will need to be extended to take into account artifacts of assembly such as component defects and differences in component stoichiometry and bond energies, this work adds to growing evidence that the physics of assembly of multicomponent, aperiodic structures is not simply an extension of principles for assembling homogeneous or periodic structures [6]. Assembly of multicomponent lattices and crystals also appear to occur far from equilibrium in general [49], [50] even when component depletion is offset by continued production of new components, as happens in *in vivo* systems. Specific attention to effects that arise in multicomponent systems, such as the interplay between combinatoric and thermodynamic factors explored here, are likely to be important in developing the capacity to self-assemble larger, more intricate structures robustly.

## Methods

### Stochastic kinetic simulations

The dynamics of the reactions to form a complex are determined using Gillespie sampling of stochastic chemical kinetics [51]. While typically stochastic fluctuations are not important to assembly results, the Gillespie algorithm makes it possible to statistically sample kinetic trajectories that would otherwise be inaccessible because the numerical integration of the coupled set of ODEs for mass action kinetics is intractable for most of the complexes we study (Table S1 and Text S2). For small complexes where comparison is possible, stochastic kinetic simulations and mass action kinetics produce nearly identical results (Fig. S21).

### Rate constants and physical parameters

For all reactions, the macroscopic on rate constant is assumed to be constant, 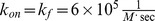, reflecting experimental data for DNA and proteins [29]–[32], which additionally simplifies analysis by providing an energy landscape for assembly. Because in practice intermediates and complexes may diffuse more slowly than components due to their increased size, this assumption likely underestimates assembly times. We define dimensionless time.
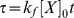
(1)where 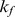 is the macroscopic on rate constant, 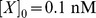 is the initial component concentration and 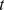 is dimensional reaction time in seconds.

To model the interplay of changes in bond energy that could result from multi-bond reactions, we introduce a dimensionless bond coupling term 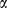 that determines how the free energy of interaction scales with the number of bonds formed. This bond coupling term is given by:
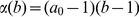
(2)where 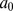 is a dimensionless coupling constant and 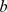 is the number of bonds formed in the reaction. Interfaces are energetically independent in the case of zero (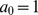) bond coupling. Negative coupling (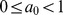) means that the interaction of multiple bonds is less favorable than the sum of the individual bond energies whereas positive coupling (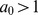) means the same interaction is more favorable than the sum of the individual bond energies. The coupling term appears in the macroscopic off rate equation:
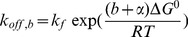
(3)where 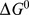 is the change in standard Gibbs free energy for a component-component interaction through a single bond, 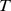 is absolute temperature and 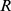 is the universal gas constant.

For detailed information on species and reaction enumeration algorithms, as well as kinetic simulation specifics, see Text S1.

## Supporting Information

Figure S1 Valid and invalid species in the 2×2 grid complex. (a) Valid species are components, full complexes and multiple component configurations, or intermediates, where all components comprising an intermediate have at least one bond (shared edge) with another component. A black box represents an occupied site whereas a white box represents unoccupied sites on the lattice. (b) Invalid intermediate assemblies are denoted by red “X”s and are lattice configurations that are not connected (do not share an edge) and are not included in our model.
(TIF)

Figure S2 Valid and invalid reactions for the 2×2 grid complex. Examples of valid reactions in a 2×2 grid complex in which (a) one bond, or (b) two bonds are formed. The reverse reaction rate (indicated roughly as arrow length) will change with reaction conditions and bond coupling. Reactions such as in (c) and (d) are not included in our model. In (c), the components do not interact at any edges and would not produce a valid species as a product, and in (d) the reactants share components in the same position, which would in practice block that reaction from happening.
(TIF)

Figure S3Yields of 1×3 to 1×9 line complexes at various isothermal conditions. Dashed lines indicate thermodynamic equilibrium. Inset diagrams depict the complexes. Here, as in the main text, 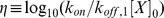 and 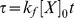. For all figures in the Supporting Information, unless otherwise noted, there is no bond coupling (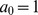) and error bars are <1%.(TIF)

Figure S4Yield of 1×9 line complex at various reaction times, 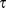, subject to different isothermal assembly conditions. Dashed lines indicate equilibrium values at a given value of 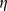. Inset diagram depicts the complex.(TIF)

Figure S5Assembly size distribution at different isothermal assembly conditions after 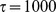. Thermodynamic equilibrium predictions are dashed lines and in all cases directly overlay the reported fractions. Inset diagrams depict the complexes.(TIF)

Figure S6Yields of 2×2, 3×3 and 4×4 square grid complexes at different isothermal assembly conditions and bond coupling constants (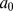). Dashed lines indicate yield at thermodynamic equilibrium. Inset diagrams depict the complexes. As bond coupling increases, intermediates and complexes become more stable (as seen by the increase in melting temperature at thermodynamic equilibrium) but nucleation rates remain approximately constant such that complex yields approach equilibrium for negative bond coupling under nucleation-limited conditions (*e.g.,
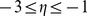
*) but remain far from equilibrium for positive bond coupling.(TIF)

Figure S7Yields of 2×2, 3×3 and 4×4 square grid complexes as a function of bond coupling constant, 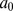, at various isothermal conditions (solid lines) and anneal (dash-dot line). Dashed lines indicate equilibrium values at the given value of 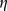. Inset diagrams depict the complexes. Above a relatively low threshold of bond coupling (whose exact value depends on assembly size and assembly conditions), assembly yields are largely insensitive to bond coupling values (see Text S5 for further explanation).(TIF)

Figure S8Yield of 3×3 square grid complex for many isothermal conditions, from 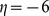 to 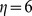 in increments 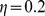. Inset diagram depicts the complex.(TIF)

Figure S9Reducing the number of components in the simulation does not significantly affect yield predictions. Yield of 2×2, 3×3 and 4×4 square grid complexes at various isothermal conditions starting with 1000 (instead of 10000) of each component, with the simulated volume adjusted so that 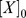 is unchanged. Dots indicate the yield of complexes at various isothermal conditions starting with 10000 of each component. Dashed line indicates yield at thermodynamic equilibrium. Inset diagrams depict the complexes(TIF)

Figure S10Yield for 2×2 and 3×3 square grid complexes at various isothermal conditions, including yield predictions after long reaction times, 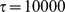. Dashed line indicates the yield at thermodynamic equilibrium. Inset diagrams depict the complexes. These results suggest that further increasing assembly time beyond what we consider in the main text does not significantly increase yields under most conditions(TIF)

Figure S11Yields of 2×2, 3×3, 4×4 and 5×5 square grid complexes at various reaction times, 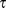, subject to different assembly conditions. Inset diagrams depict the complexes. Dashed lines correspond to thermodynamic equilibrium and color corresponds to the value of 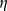. Dash-dot line connects complex yields of anneals with various reaction times, 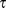. For 2×2, 3×3 and 4×4 square grid complexes, 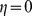 is within the assembly funnel regime, but for the 5×5 complex 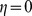 is within the parallel pathways and rearrangement-limited regime.(TIF)

Figure S12Yield of 2×2, 3×3 and 4×4 spiral complexes at various reaction times, 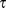, subject to different assembly conditions. Inset diagrams depict the complexes. Dash-dot line connects complex yields after anneals with various reaction times, 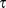.(TIF)

Figure S13Assembly size distributions (in # of components) for 2×2, 3×3 and 4×4 square grid complexes at various isothermal conditions and bond coupling constants. Inset diagrams depict the complexes. All plots are shown after 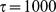
_._
(TIF)

Figure S14Timescales of nucleation and rearrangement together determine the rate of complex formation. Both of these timescales are functions of complex size and geometry. The fraction of material in various species as a function of reaction time for 3×3 square grid assembly under different assembly regimes: nucleation-limited at 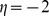, assembly funnel regime at 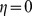 and rearrangement-limited at 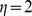. Inset diagram depicts a possible reaction pathway for nucleation and arrow size indicates relative reaction propensities.(TIF)

Figure S15Size distribution of intermediates for various 2D complexes. Mean size of intermediates (in number of components) after 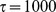, 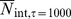, normalized by the number of components in the complex, 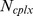, for 2×2, 3×3, 4×4 and 5×5 square grid complexes at different isothermal assembly conditions. Inset diagrams depict the complexes. The mean intermediate size is defined as the mean size of the species in the system, not including complexes or components. Nucleation-limited conditions produce mean intermediate assembly sizes equal or less than half of the size of a complex whereas rearrangement-limited conditions allow intermediates to grow to be, on average, greater than half of the size of a complex.(TIF)

Figure S16During an anneal, most complexes are produced during the phase of the anneal that passes through the assembly funnel. Yield of 2×2 and 3×3 square grid complexes during the course of an anneal for various bond coupling constants. Inset diagrams depict the complexes being assembled. The anneal begins from left to right, with the total time of the anneal given as the value of 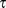 in the legend. The annealing process is simulated by changing the strength of component-component interactions as the reaction proceeds. At the start of the simulation (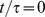), 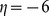 and over the course of the simulation the interaction strength is logarithmically increased 100 times, in equal reaction time intervals (i.e., 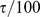), to ultimately obtain 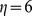 at the end of the simulation (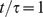). In practice, this annealing protocol corresponds to a linear decrease in temperature over time. Assembly regimes are determined by isothermal assembly (see Figure S6).(TIF)

Figure S17During very long anneals, component depletion can increase the amount of time that the system effectively stays within the assembly funnel regime. Effective reaction propensity is given by 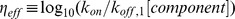 where 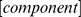 is the current average component concentration, for the 2×2 and 3×3 square grid complexes as a function of annealing conditions after various annealing times. Color bars on the left side of the figures correspond to different assembly regimes. Inset diagrams depict the complexes. Effective reaction propensities for slower anneals remain in the assembly funnel regime for longer periods of time, not only because of their increased time of anneal, but also because components are depleted during annealing. This decrease offsets the effect of the off rate (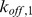) decreasing as the temperature decreases. As a result, during a slow anneal 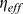 can be in the assembly funnel regime even as 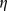 drops into rearrangement-limited conditions. During fast anneals (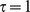), the off rate changes much faster than components deplete, accounting for the linear relationship between 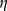 and 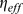. Dashed line approximates the 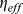 for an ideal anneal (where 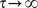). In an ideal anneal, components would deplete in proportion to the decrease in the off rate and thus always remain in the assembly funnel regime after initially entering it.(TIF)

Figure S18The time spent in the assembly funnel regime can be used to predict the outcome of an anneal**.** Yield of 3×3 square grid complex as a function of reaction time for an isothermal assembly (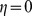) and for an anneal. Inset diagram depicts the complex. For a 3×3 square grid complex, the assembly funnel regime ranges from 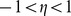 (see Figure 2). The red and blue dots are estimated yields calculated by computing the time the anneal spends in the assembly funnel regime and, with this value, estimating yield by linear interpolation of an 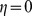 isothermal assembly. With no component depletion effects (red), a given anneal of time 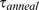, will spend 
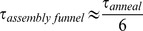
 in the assembly funnel regime. With component depletion effects (blue, see Figure S16), the time spent in the assembly funnel regime will correspond to the time that the anneal remained 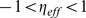 so that the slower the anneal, the higher the fraction of total reaction time spent in the assembly funnel regime. For example, when 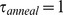, 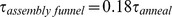 and when 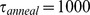, 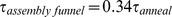. The method of estimating yield via annealing that includes component depletion effects more closely resembles the actual annealing yield, suggesting that component depletion effects, which serve to increase the time spent in the assembly funnel regime and in turn enhance yields, occurs during annealing.(TIF)

Figure S19 Deterministic and stochastic solutions are almost identical. To test the similarity of the stochastic solution to the deterministic solution, we simulated the ODEs for the respective complexes using MATLAB’s ode23s solver. Deterministic solution (solid lines) and overlaid stochastically sampled values (dots) of yield for 2×2 and 3×3 square grid and 2×2 spiral complexes at various isothermal conditions. Inset diagrams depict complexes.
(TIF)

Figure S20Yields of 2×2x2 cube complexes as a function of bond coupling constants 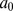 at various isothermal conditions. Dashed line indicates complex yield at thermodynamic equilibrium. Inset diagram depicts the complex.(TIF)

Figure S21Yield of 2×2x2 cube complexes as a function of bond coupling constant, 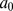 at various isothermal conditions (in terms of 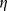). Dashed lines indicate equilibrium values at the given value of 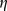. Inset diagram depicts the complex.(TIF)

Figure S22Yield of 2×2x2 cube complex at various reaction times, 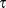, subject to different isothermal assembly conditions. Dashed lines indicate equilibrium values of yield at the given value of 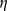 (equilibrium yield is unity for all values of 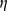 shown). Inset diagram depicts the complex.(TIF)

Figure S23Assembly size distributions for 2×2x2 cube complex at various isothermal conditions and bond coupling constants. All plots are shown after 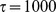. Inset diagram depicts the complex.(TIF)

Table S1 Complex specifics and parameter space explored in this work.
(DOCX)

Table S2 Criteria for labeling assembly regimes.
(DOCX)

Text S1 Supporting Methods.
(DOCX)

Text S2 Computational Specifics.
(DOCX)

Text S3 Assembly Regime Criteria.
(DOCX)

Text S4 Assembly Distribution Selection.
(DOCX)

Text S5 Further Explanation of Bond Coupling Effect on Yield (from Figure 5).
(DOCX)

Text S6 Computing Thermodynamic Equilibrium of Large Complexes.
(DOCX)

Text S7 Supporting References.
(DOCX)
