# Supplementary figures and images for: An Assembly Funnel Makes Biomolecular Complex Assembly Efficient
Source: PLoS One. 2014 Oct 31;9(10):e111233. doi: 10.1371/journal.pone.0111233 (PMC4215988; doi:10.1371/journal.pone.0111233)

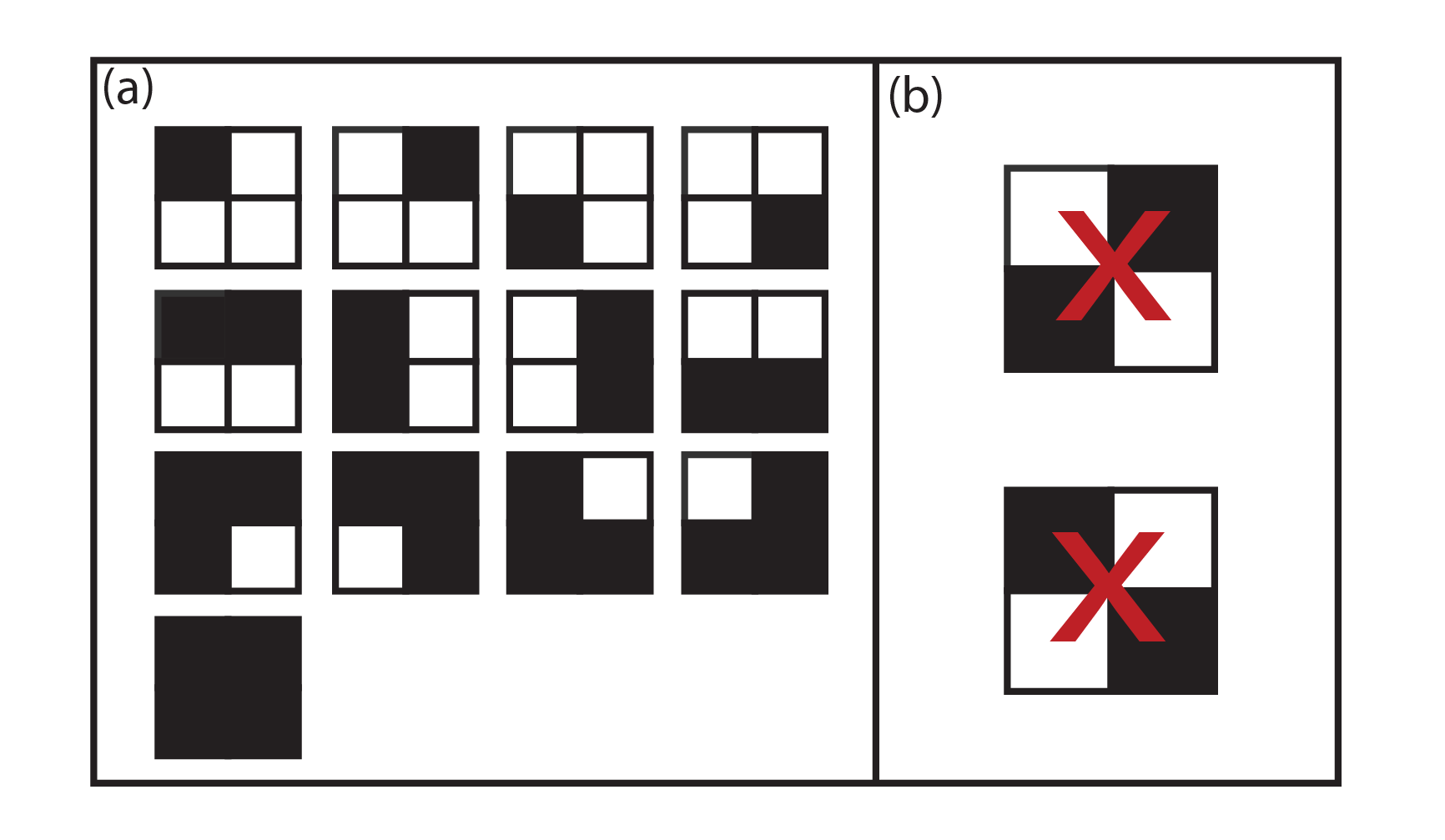

Supplement: Figure S1 — Valid and invalid species in the 2×2 grid complex. (a) Valid species are components, full complexes and multiple component configurations, or intermediates, where all components comprising an intermediate have at least one bond (shared edge) with another component. A black box represents an occupied site whereas a white box represents unoccupied sites on the lattice. (b) Invalid intermediate assemblies are denoted by red “X”s and are lattice configurations that are not connected (do not share an edge) and are not included in our model. (TIF) [file pone.0111233.s001.tif]

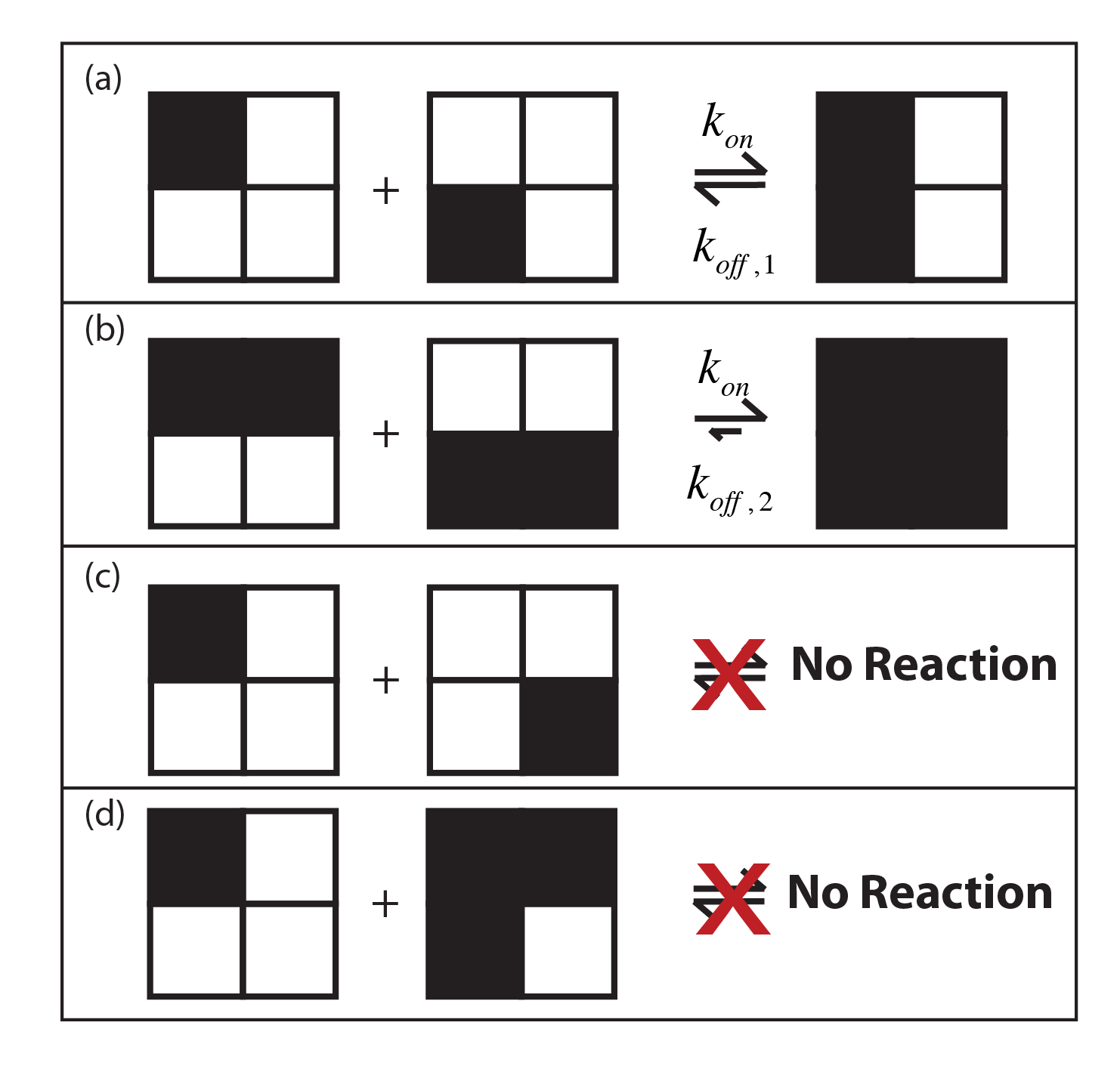

Supplement: Figure S2 — Valid and invalid reactions for the 2×2 grid complex. Examples of valid reactions in a 2×2 grid complex in which (a) one bond, or (b) two bonds are formed. The reverse reaction rate (indicated roughly as arrow length) will change with reaction conditions and bond coupling. Reactions such as in (c) and (d) are not included in our model. In (c), the components do not interact at any edges and would not produce a valid species as a product, and in (d) the reactants share components in the same position, which would in practice block that reaction from happening. (TIF) [file pone.0111233.s002.tif]

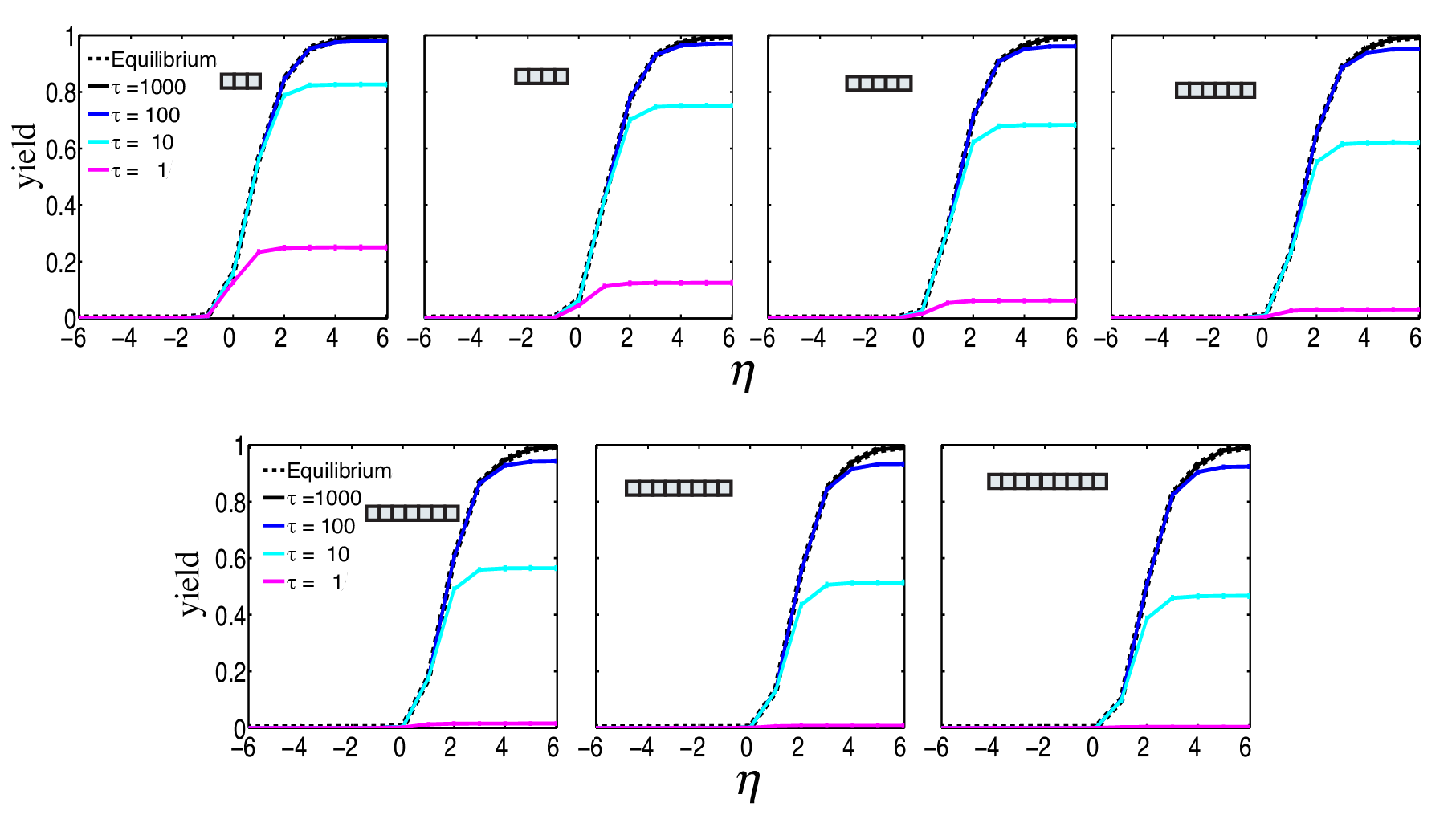

Supplement: Figure S3 — Yields of 1×3 to 1×9 line complexes at various isothermal conditions. Dashed lines indicate thermodynamic equilibrium. Inset diagrams depict the complexes. Here, as in the main text, and . For all figures in the Supporting Information, unless otherwise noted, there is no bond coupling () and error bars are <1%. (TIF) [file pone.0111233.s003.tif]

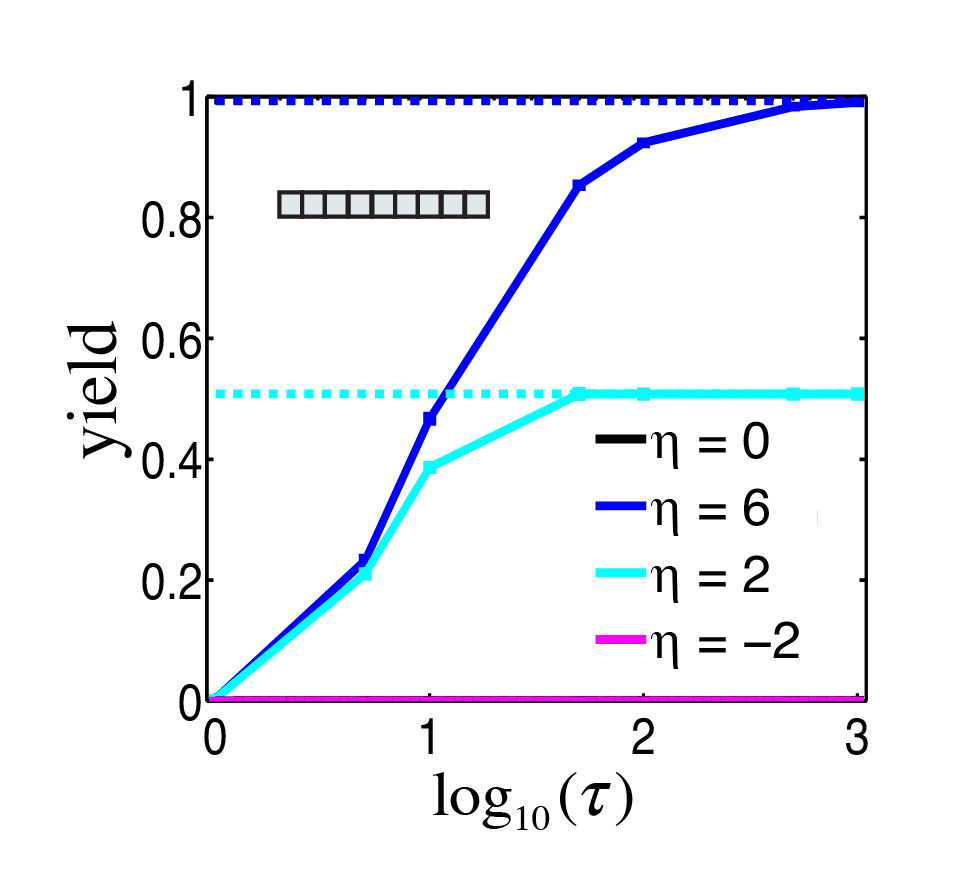

Supplement: Figure S4 — Yield of 1×9 line complex at various reaction times, , subject to different isothermal assembly conditions. Dashed lines indicate equilibrium values at a given value of . Inset diagram depicts the complex. (TIF) [file pone.0111233.s004.tif]

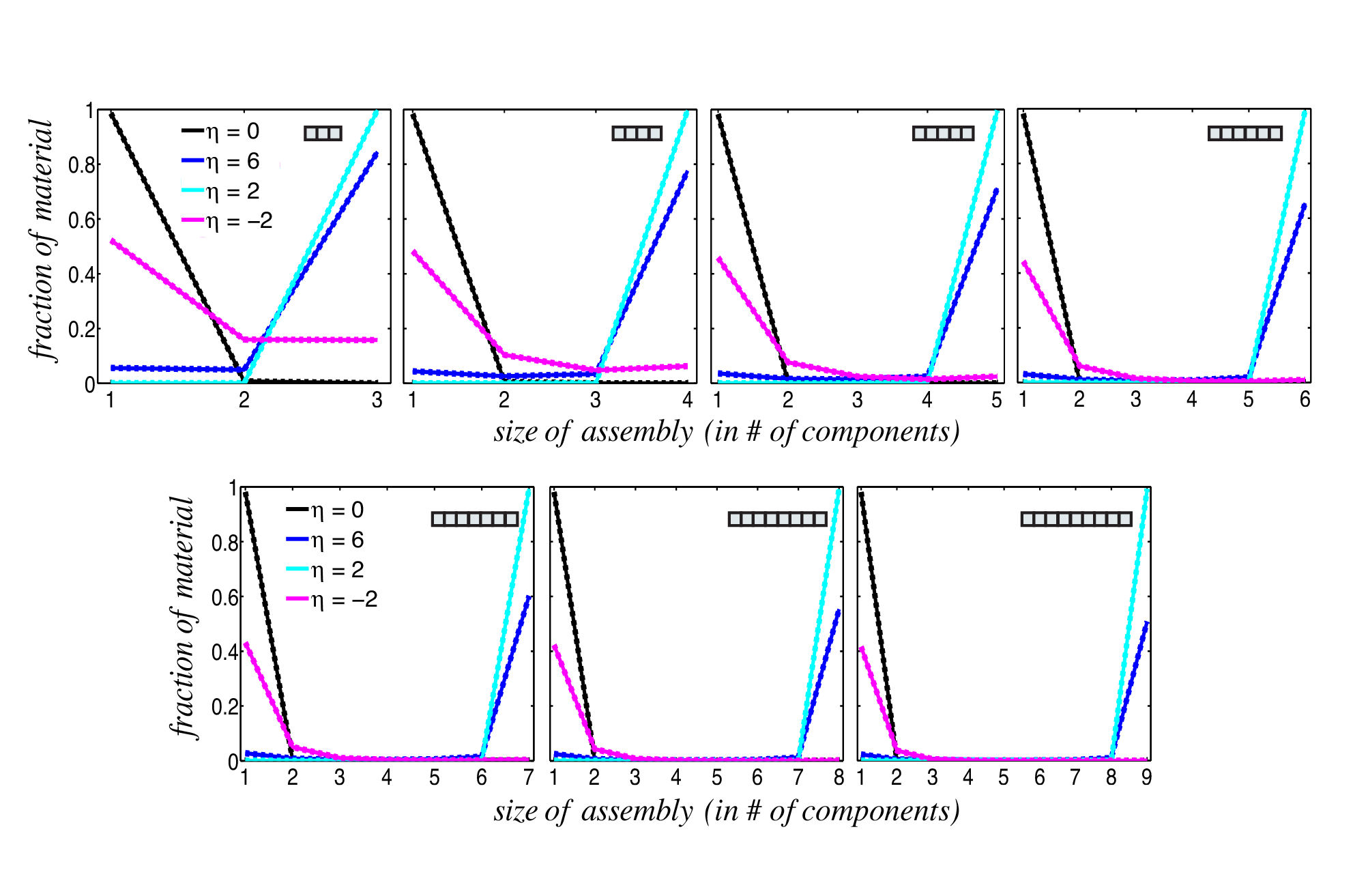

Supplement: Figure S5 — Assembly size distribution at different isothermal assembly conditions after . Thermodynamic equilibrium predictions are dashed lines and in all cases directly overlay the reported fractions. Inset diagrams depict the complexes. (TIF) [file pone.0111233.s005.tif]

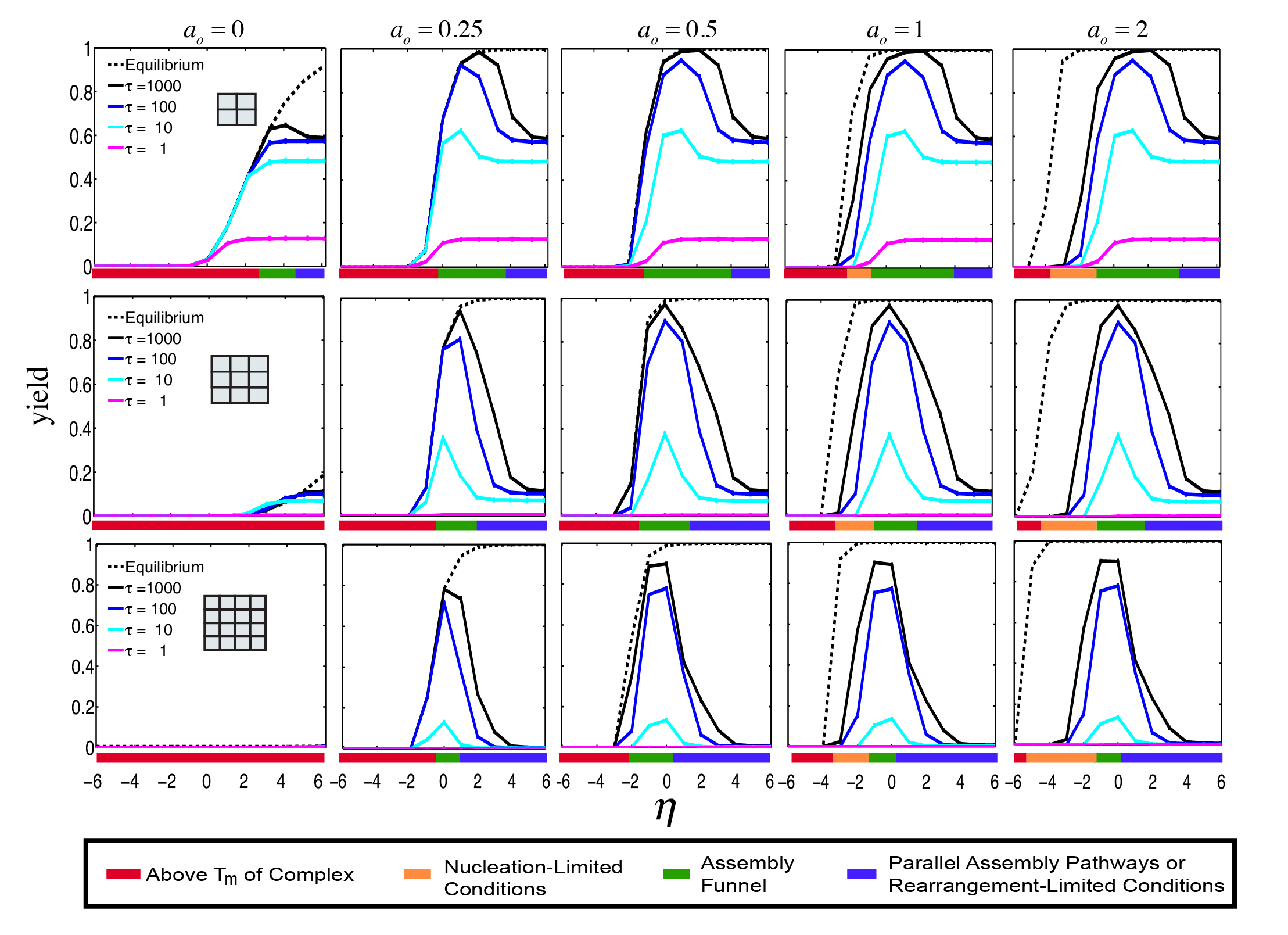

Supplement: Figure S6 — Yields of 2×2, 3×3 and 4×4 square grid complexes at different isothermal assembly conditions and bond coupling constants (). Dashed lines indicate yield at thermodynamic equilibrium. Inset diagrams depict the complexes. As bond coupling increases, intermediates and complexes become more stable (as seen by the increase in melting temperature at thermodynamic equilibrium) but nucleation rates remain approximately constant such that complex yields approach equilibrium for negative bond coupling under nucleation-limited conditions (e.g.,) but remain far from equilibrium for positive bond coupling. (TIF) [file pone.0111233.s006.tif]

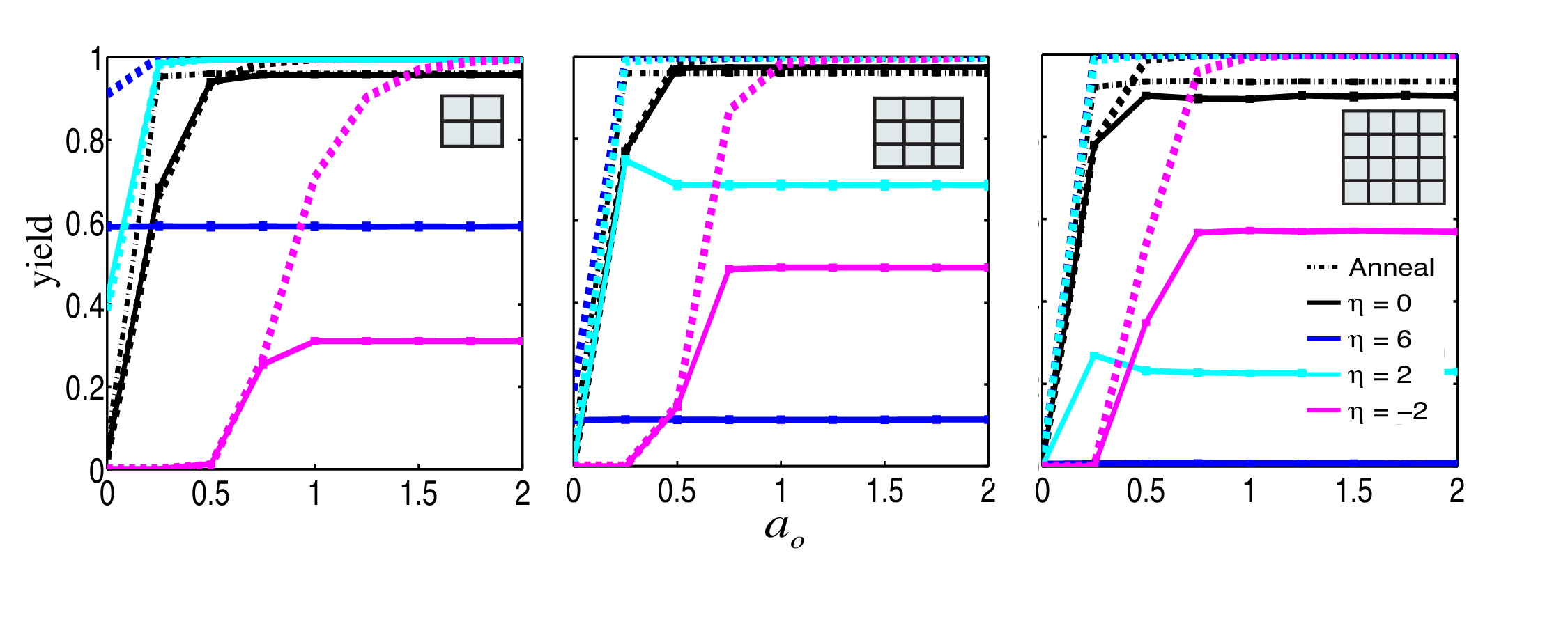

Supplement: Figure S7 — Yields of 2×2, 3×3 and 4×4 square grid complexes as a function of bond coupling constant, , at various isothermal conditions (solid lines) and anneal (dash-dot line). Dashed lines indicate equilibrium values at the given value of . Inset diagrams depict the complexes. Above a relatively low threshold of bond coupling (whose exact value depends on assembly size and assembly conditions), assembly yields are largely insensitive to bond coupling values (see Text S5 for further explanation). (TIF) [file pone.0111233.s007.tif]

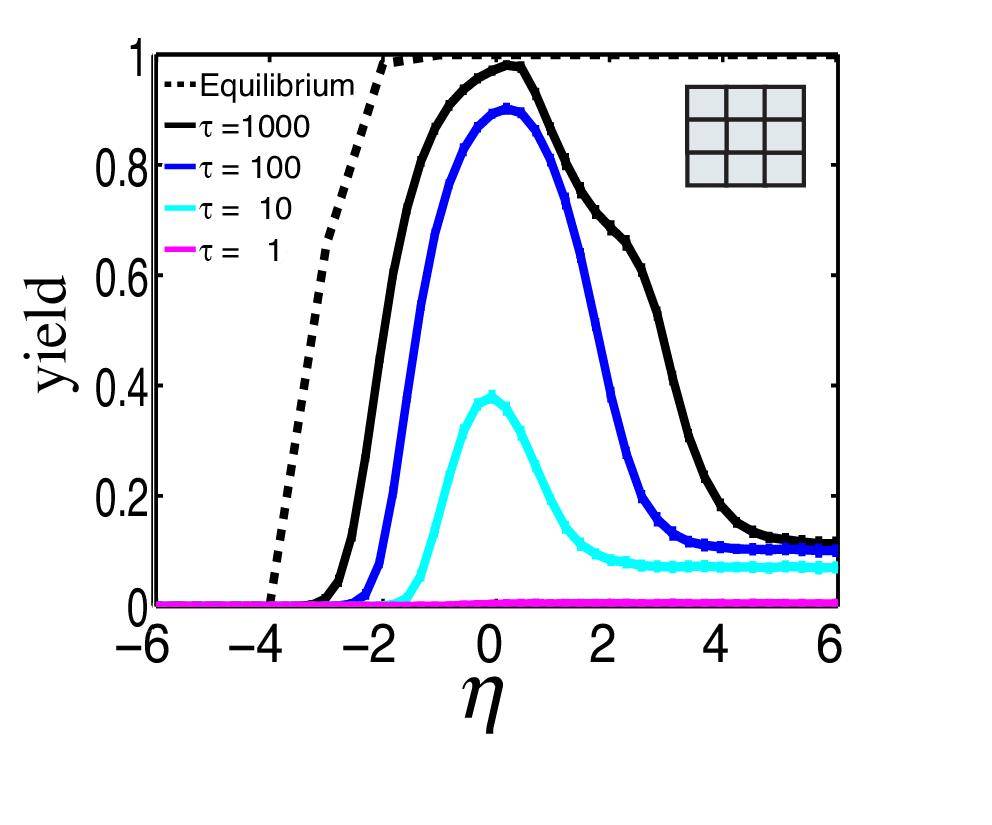

Supplement: Figure S8 — Yield of 3×3 square grid complex for many isothermal conditions, from to in increments . Inset diagram depicts the complex. (TIF) [file pone.0111233.s008.tif]

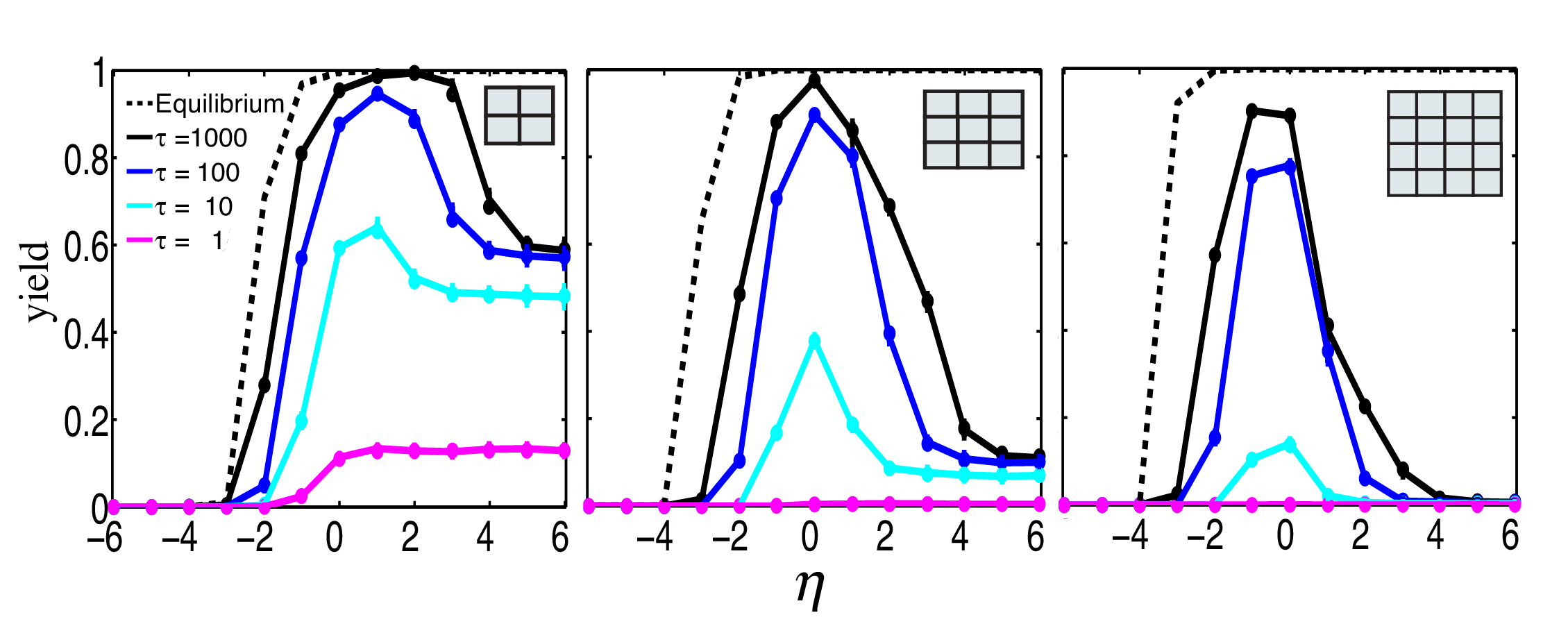

Supplement: Figure S9 — Reducing the number of components in the simulation does not significantly affect yield predictions. Yield of 2×2, 3×3 and 4×4 square grid complexes at various isothermal conditions starting with 1000 (instead of 10000) of each component, with the simulated volume adjusted so that is unchanged. Dots indicate the yield of complexes at various isothermal conditions starting with 10000 of each component. Dashed line indicates yield at thermodynamic equilibrium. Inset diagrams depict the complexes (TIF) [file pone.0111233.s009.tif]

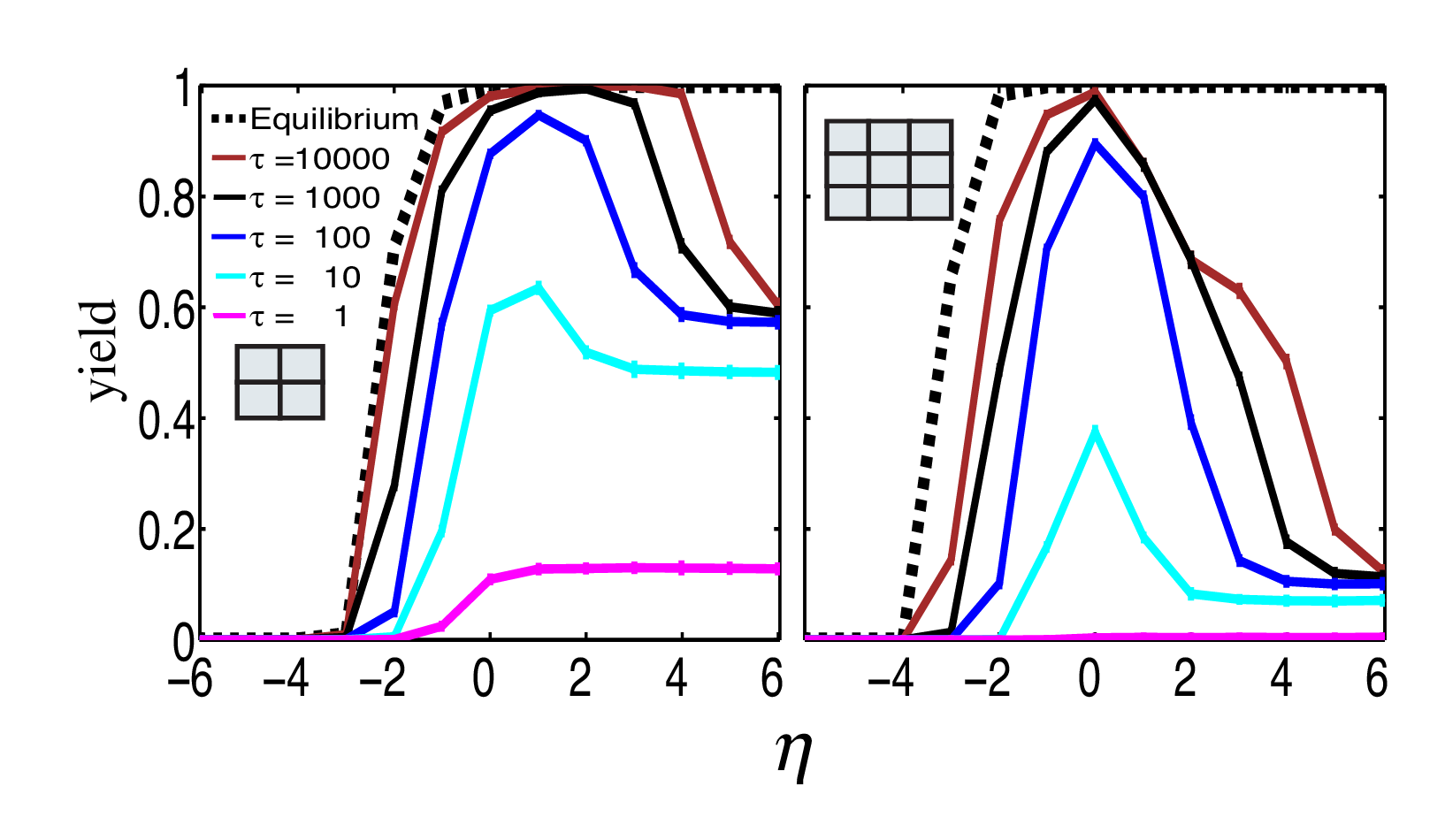

Supplement: Figure S10 — Yield for 2×2 and 3×3 square grid complexes at various isothermal conditions, including yield predictions after long reaction times, . Dashed line indicates the yield at thermodynamic equilibrium. Inset diagrams depict the complexes. These results suggest that further increasing assembly time beyond what we consider in the main text does not significantly increase yields under most conditions (TIF) [file pone.0111233.s010.tif]

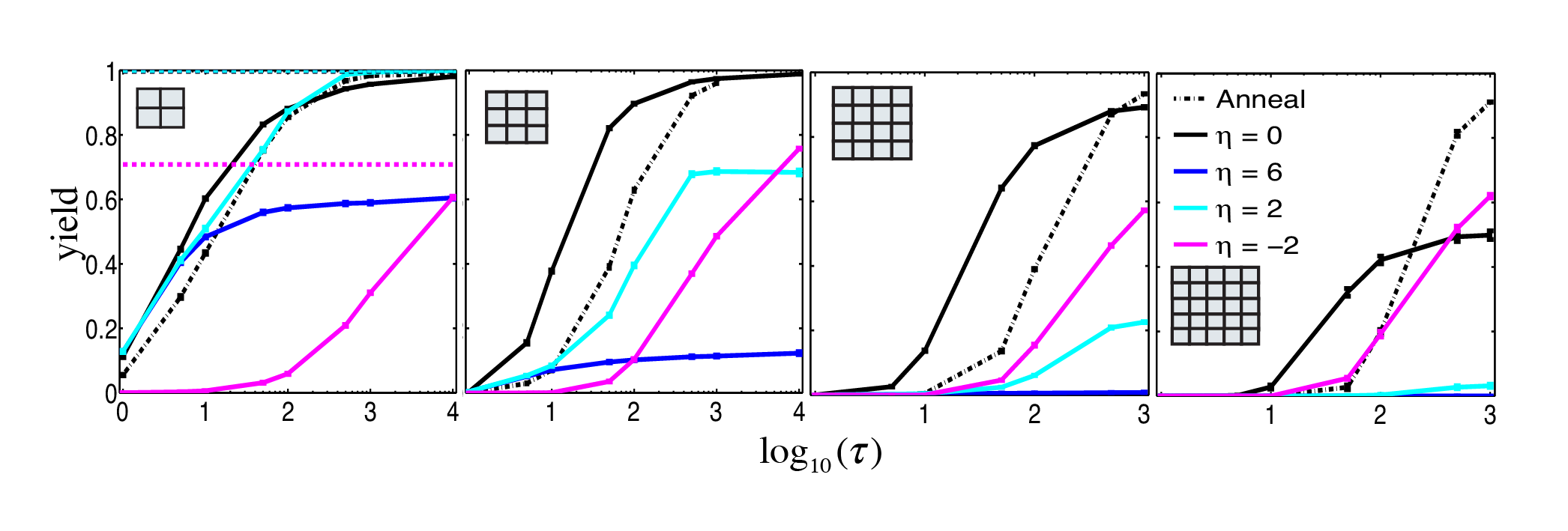

Supplement: Figure S11 — Yields of 2×2, 3×3, 4×4 and 5×5 square grid complexes at various reaction times, , subject to different assembly conditions. Inset diagrams depict the complexes. Dashed lines correspond to thermodynamic equilibrium and color corresponds to the value of . Dash-dot line connects complex yields of anneals with various reaction times, . For 2×2, 3×3 and 4×4 square grid complexes, is within the assembly funnel regime, but for the 5×5 complex is within the parallel pathways and rearrangement-limited regime. (TIF) [file pone.0111233.s011.tif]

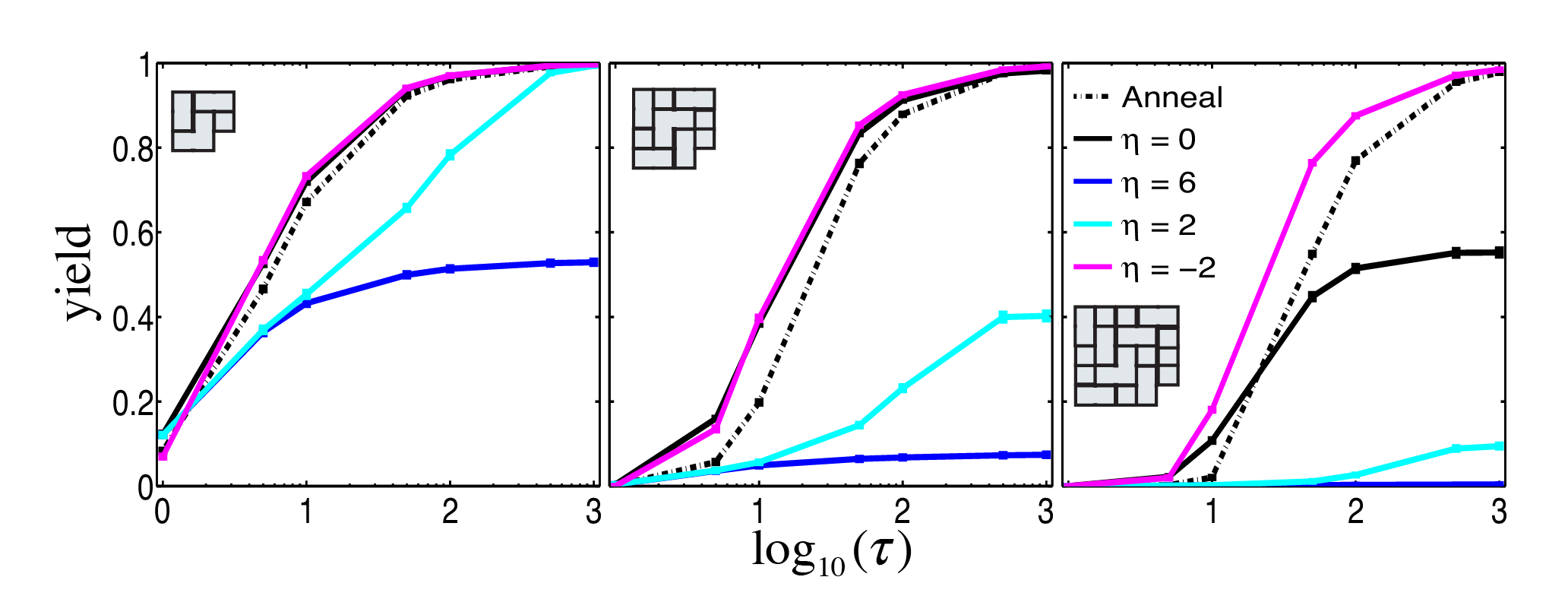

Supplement: Figure S12 — Yield of 2×2, 3×3 and 4×4 spiral complexes at various reaction times, , subject to different assembly conditions. Inset diagrams depict the complexes. Dash-dot line connects complex yields after anneals with various reaction times, . (TIF) [file pone.0111233.s012.tif]

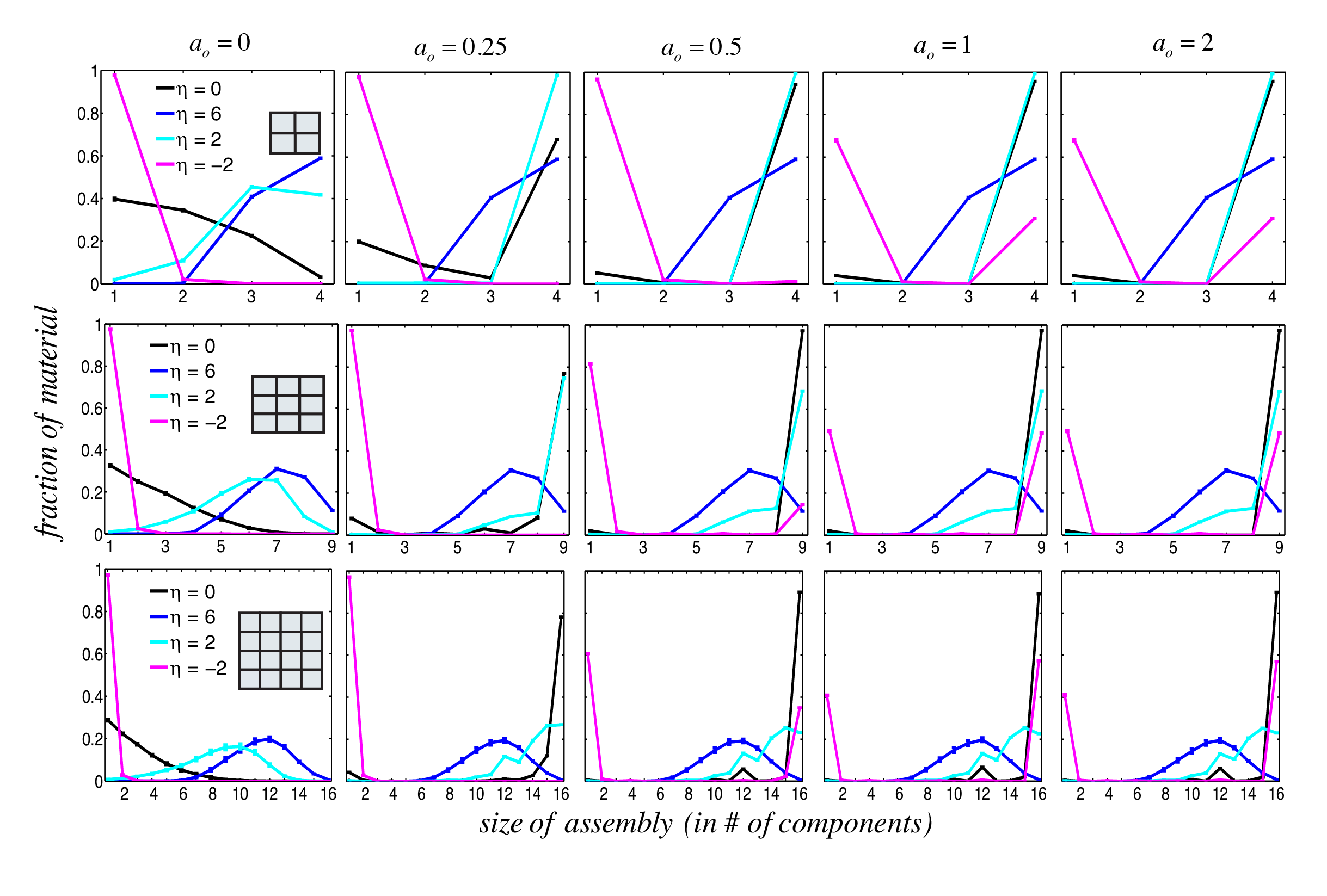

Supplement: Figure S13 — Assembly size distributions (in # of components) for 2×2, 3×3 and 4×4 square grid complexes at various isothermal conditions and bond coupling constants. Inset diagrams depict the complexes. All plots are shown after . (TIF) [file pone.0111233.s013.tif]

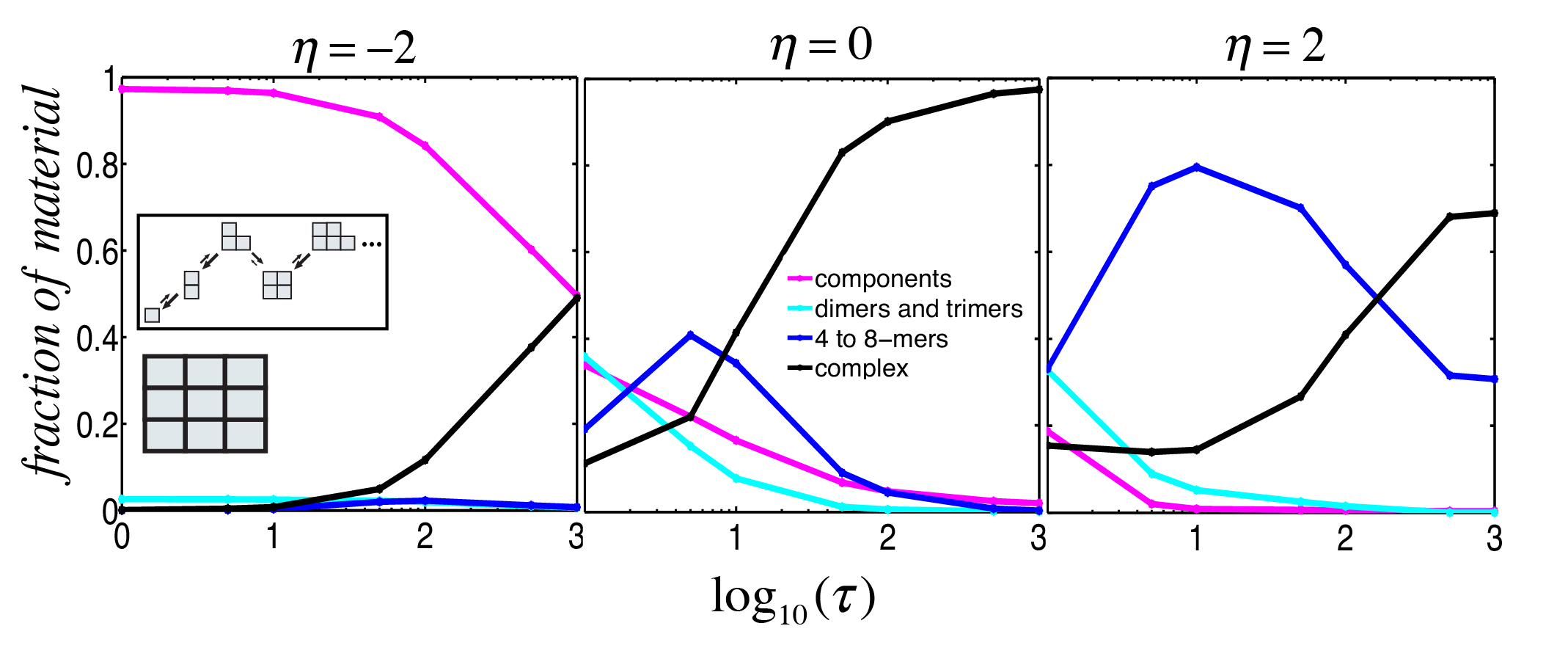

Supplement: Figure S14 — Timescales of nucleation and rearrangement together determine the rate of complex formation. Both of these timescales are functions of complex size and geometry. The fraction of material in various species as a function of reaction time for 3×3 square grid assembly under different assembly regimes: nucleation-limited at , assembly funnel regime at and rearrangement-limited at . Inset diagram depicts a possible reaction pathway for nucleation and arrow size indicates relative reaction propensities. (TIF) [file pone.0111233.s014.tif]

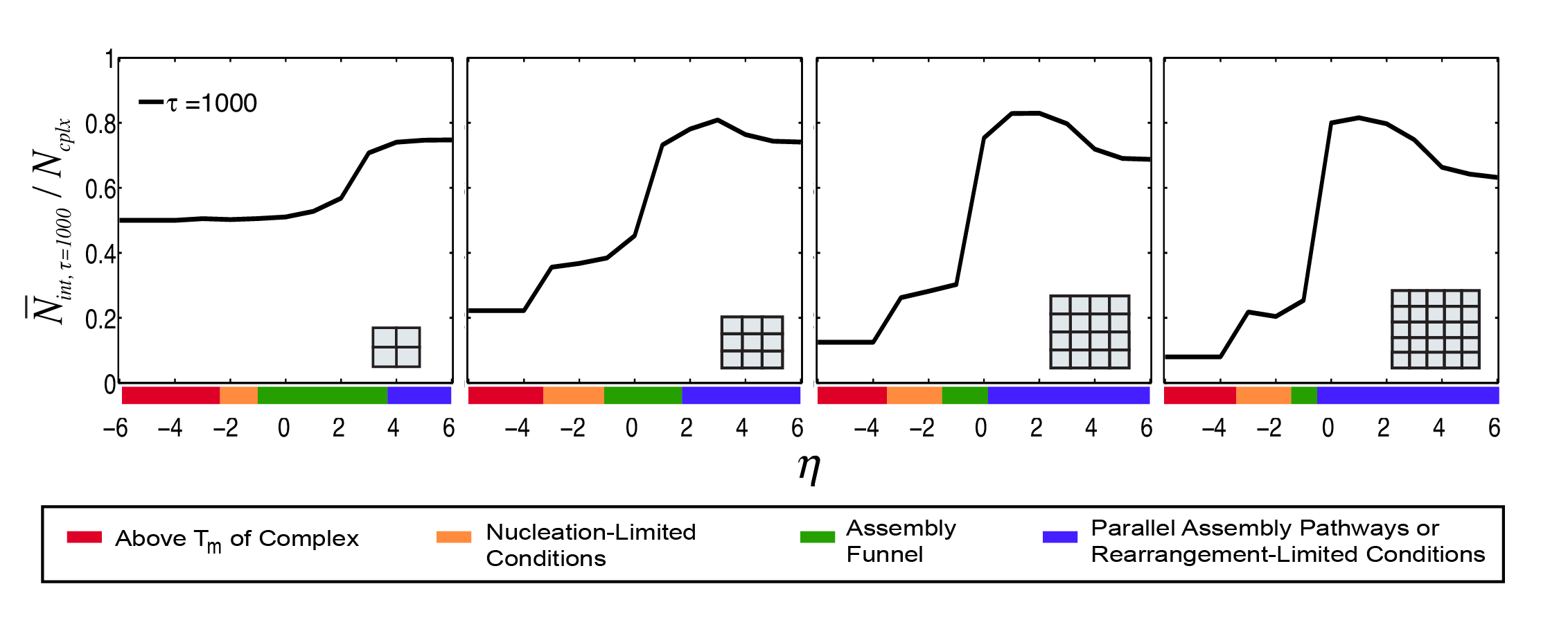

Supplement: Figure S15 — Size distribution of intermediates for various 2D complexes. Mean size of intermediates (in number of components) after , , normalized by the number of components in the complex, , for 2×2, 3×3, 4×4 and 5×5 square grid complexes at different isothermal assembly conditions. Inset diagrams depict the complexes. The mean intermediate size is defined as the mean size of the species in the system, not including complexes or components. Nucleation-limited conditions produce mean intermediate assembly sizes equal or less than half of the size of a complex whereas rearrangement-limited conditions allow intermediates to grow to be, on average, greater than half of the size of a complex. (TIF) [file pone.0111233.s015.tif]

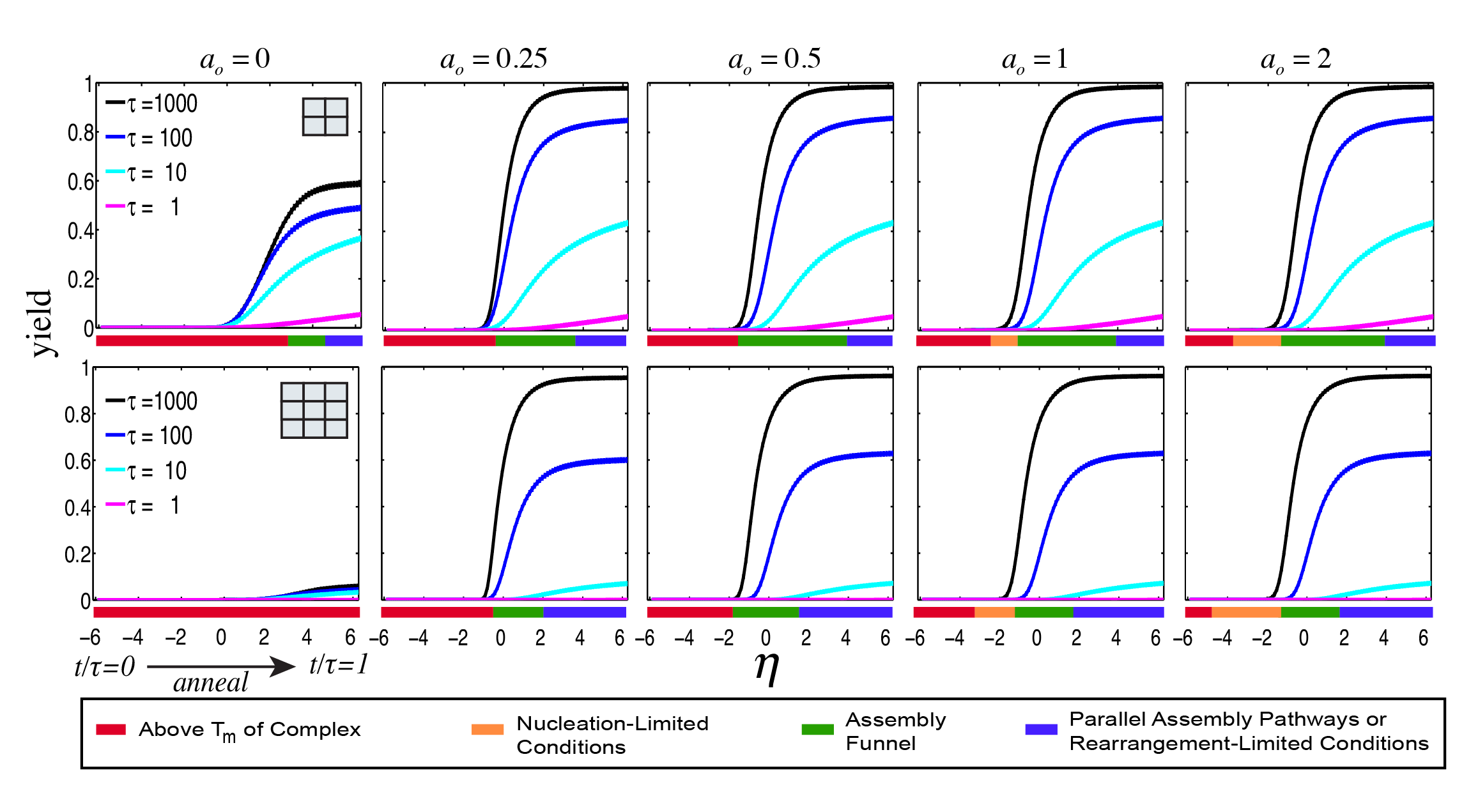

Supplement: Figure S16 — During an anneal, most complexes are produced during the phase of the anneal that passes through the assembly funnel. Yield of 2×2 and 3×3 square grid complexes during the course of an anneal for various bond coupling constants. Inset diagrams depict the complexes being assembled. The anneal begins from left to right, with the total time of the anneal given as the value of in the legend. The annealing process is simulated by changing the strength of component-component interactions as the reaction proceeds. At the start of the simulation (), and over the course of the simulation the interaction strength is logarithmically increased 100 times, in equal reaction time intervals (i.e., ), to ultimately obtain at the end of the simulation (). In practice, this annealing protocol corresponds to a linear decrease in temperature over time. Assembly regimes are determined by isothermal assembly (see Figure S6). (TIF) [file pone.0111233.s016.tif]

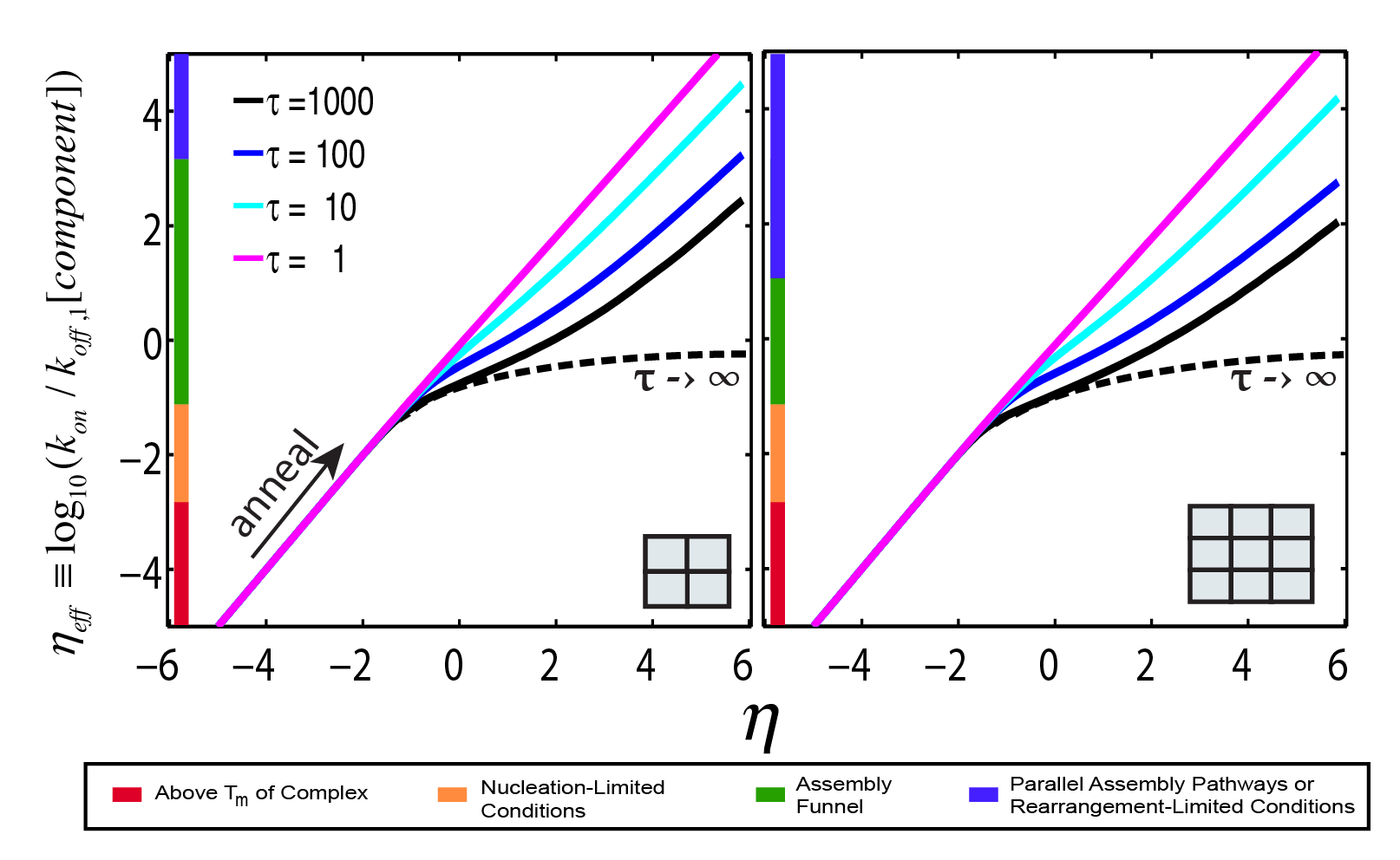

Supplement: Figure S17 — During very long anneals, component depletion can increase the amount of time that the system effectively stays within the assembly funnel regime. Effective reaction propensity is given by where is the current average component concentration, for the 2×2 and 3×3 square grid complexes as a function of annealing conditions after various annealing times. Color bars on the left side of the figures correspond to different assembly regimes. Inset diagrams depict the complexes. Effective reaction propensities for slower anneals remain in the assembly funnel regime for longer periods of time, not only because of their increased time of anneal, but also because components are depleted during annealing. This decrease offsets the effect of the off rate () decreasing as the temperature decreases. As a result, during a slow anneal can be in the assembly funnel regime even as drops into rearrangement-limited conditions. During fast anneals (), the off rate changes much faster than components deplete, accounting for the linear relationship between and . Dashed line approximates the for an ideal anneal (where ). In an ideal anneal, components would deplete in proportion to the decrease in the off rate and thus always remain in the assembly funnel regime after initially entering it. (TIF) [file pone.0111233.s017.tif]

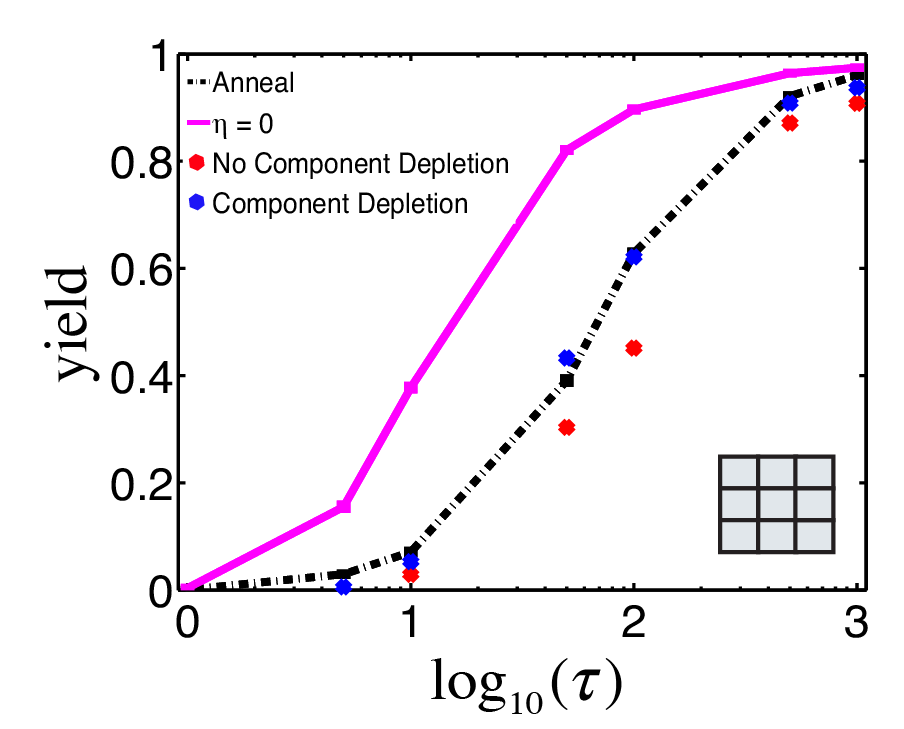

Supplement: Figure S18 — The time spent in the assembly funnel regime can be used to predict the outcome of an anneal. Yield of 3×3 square grid complex as a function of reaction time for an isothermal assembly () and for an anneal. Inset diagram depicts the complex. For a 3×3 square grid complex, the assembly funnel regime ranges from (see Figure 2). The red and blue dots are estimated yields calculated by computing the time the anneal spends in the assembly funnel regime and, with this value, estimating yield by linear interpolation of an isothermal assembly. With no component depletion effects (red), a given anneal of time , will spend in the assembly funnel regime. With component depletion effects (blue, see Figure S16), the time spent in the assembly funnel regime will correspond to the time that the anneal remained so that the slower the anneal, the higher the fraction of total reaction time spent in the assembly funnel regime. For example, when , and when , . The method of estimating yield via annealing that includes component depletion effects more closely resembles the actual annealing yield, suggesting that component depletion effects, which serve to increase the time spent in the assembly funnel regime and in turn enhance yields, occurs during annealing. (TIF) [file pone.0111233.s018.tif]

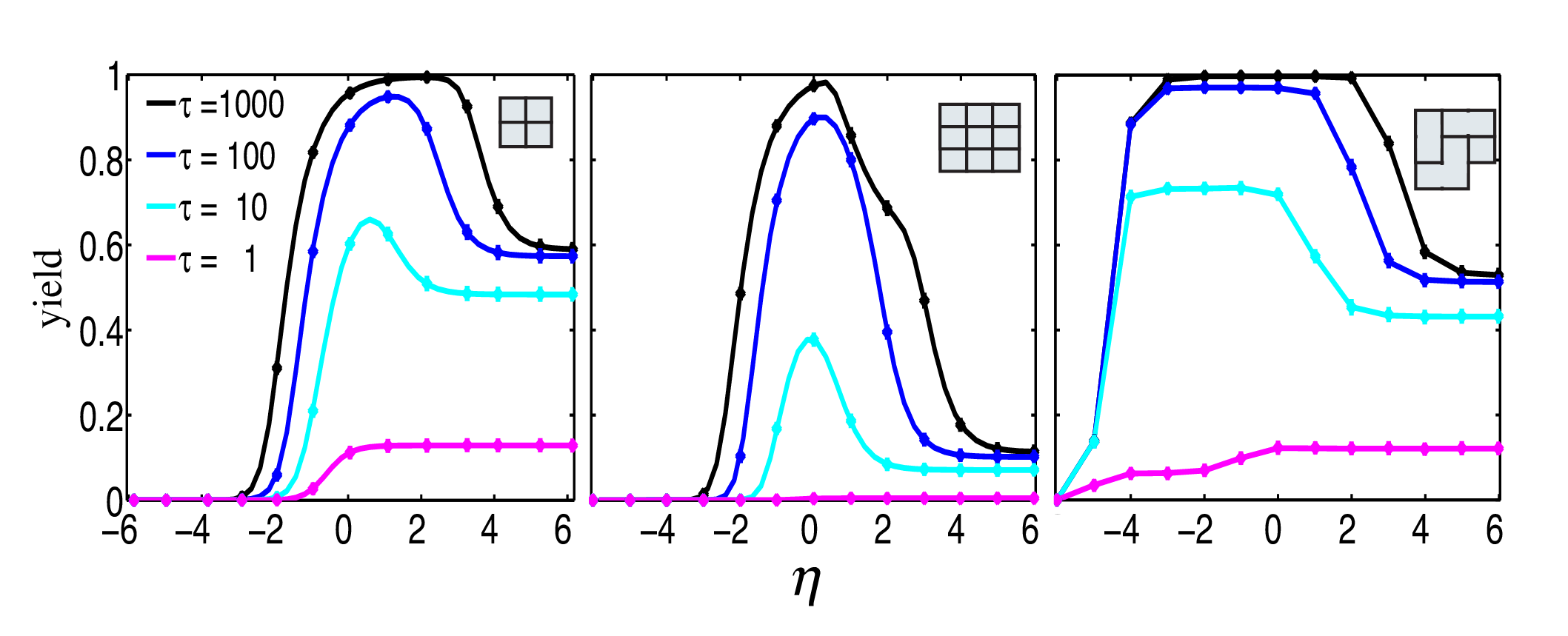

Supplement: Figure S19 — Deterministic and stochastic solutions are almost identical. To test the similarity of the stochastic solution to the deterministic solution, we simulated the ODEs for the respective complexes using MATLAB’s ode23s solver. Deterministic solution (solid lines) and overlaid stochastically sampled values (dots) of yield for 2×2 and 3×3 square grid and 2×2 spiral complexes at various isothermal conditions. Inset diagrams depict complexes. (TIF) [file pone.0111233.s019.tif]

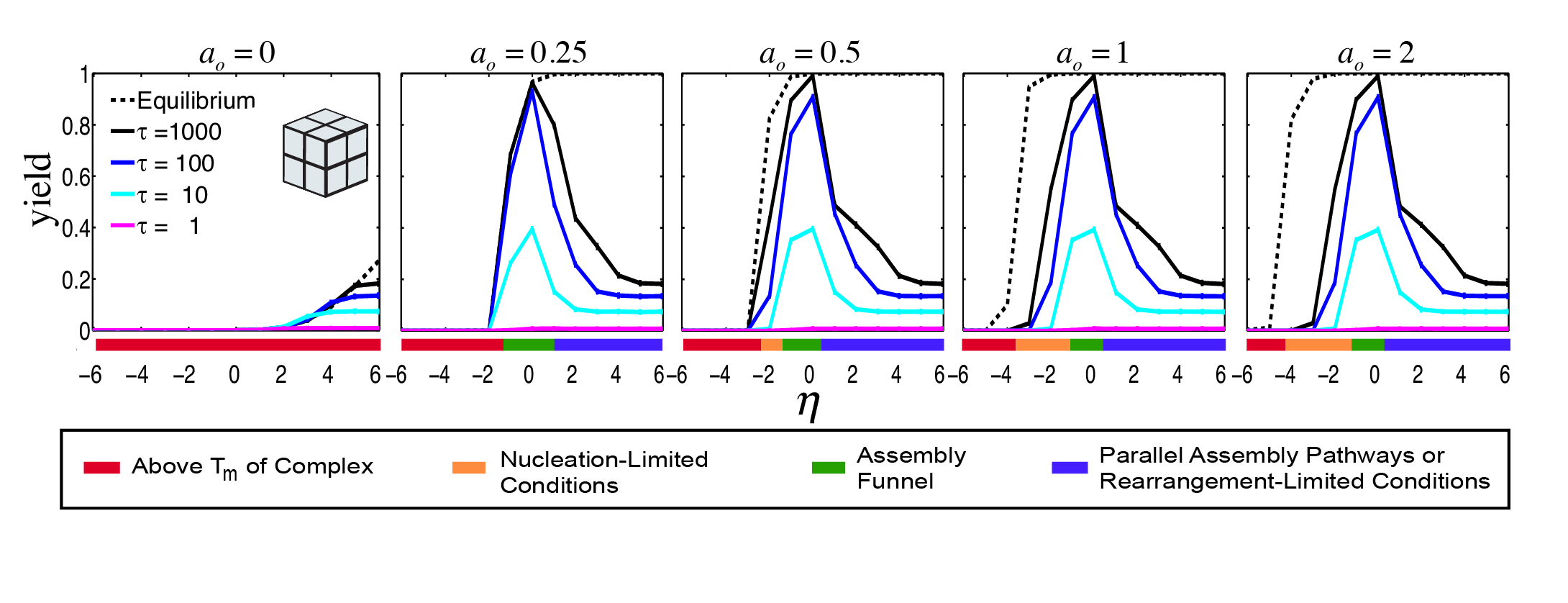

Supplement: Figure S20 — Yields of 2×2x2 cube complexes as a function of bond coupling constants at various isothermal conditions. Dashed line indicates complex yield at thermodynamic equilibrium. Inset diagram depicts the complex. (TIF) [file pone.0111233.s020.tif]

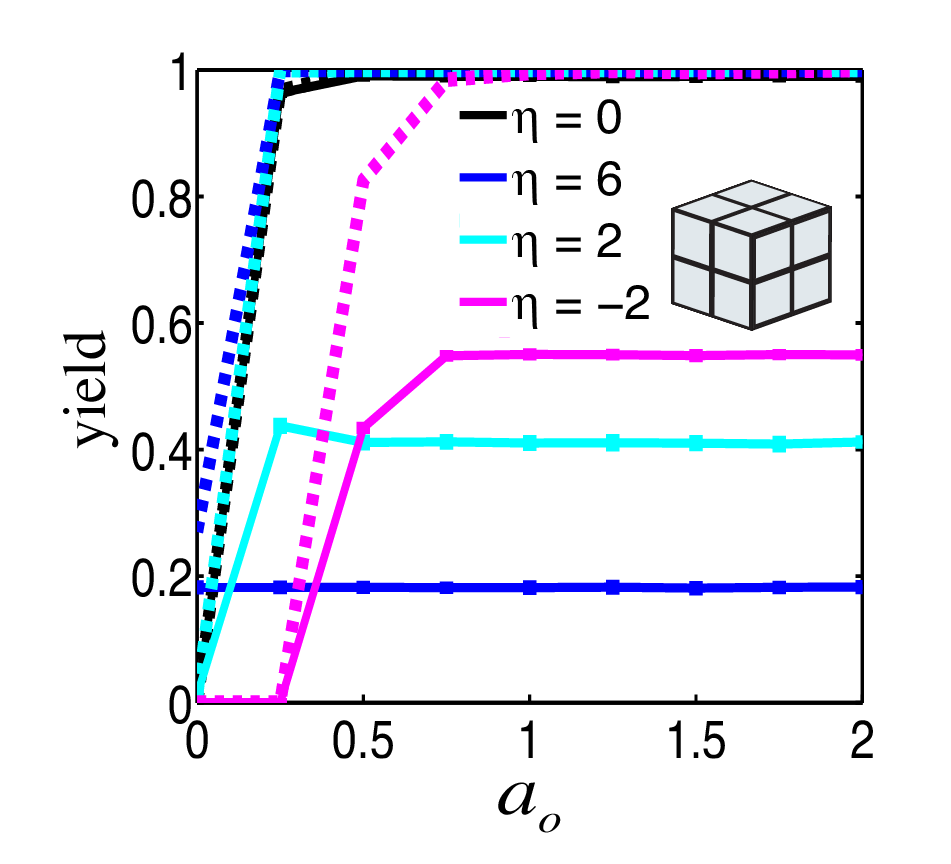

Supplement: Figure S21 — Yield of 2×2x2 cube complexes as a function of bond coupling constant, at various isothermal conditions (in terms of ). Dashed lines indicate equilibrium values at the given value of . Inset diagram depicts the complex. (TIF) [file pone.0111233.s021.tif]

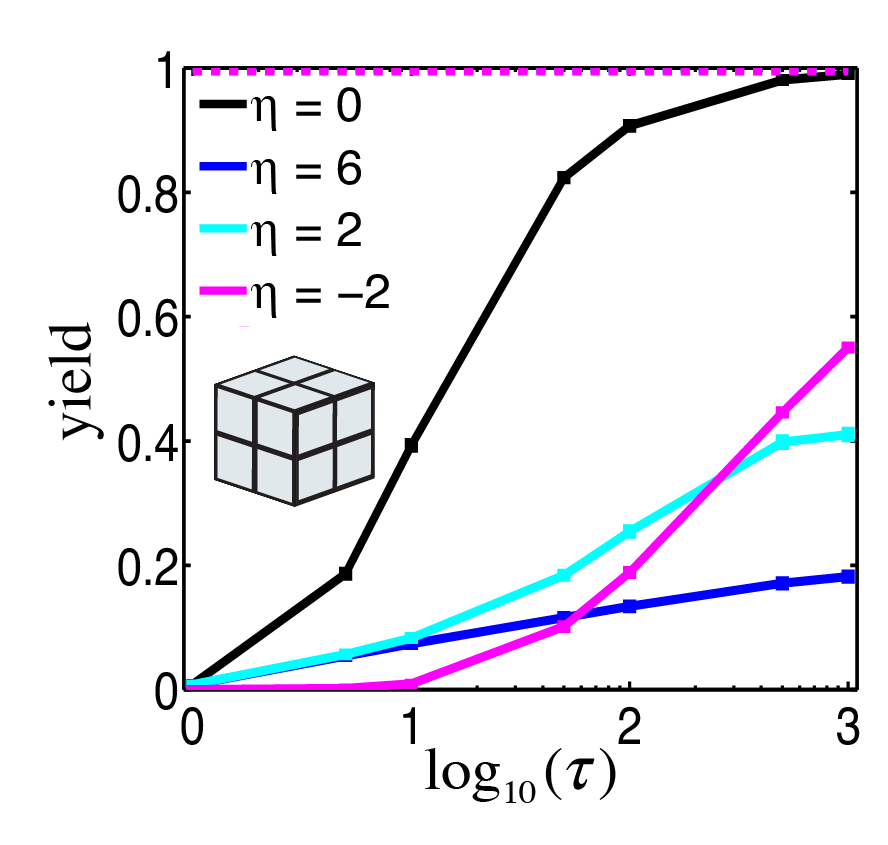

Supplement: Figure S22 — Yield of 2×2x2 cube complex at various reaction times, , subject to different isothermal assembly conditions. Dashed lines indicate equilibrium values of yield at the given value of (equilibrium yield is unity for all values of shown). Inset diagram depicts the complex. (TIF) [file pone.0111233.s022.tif]

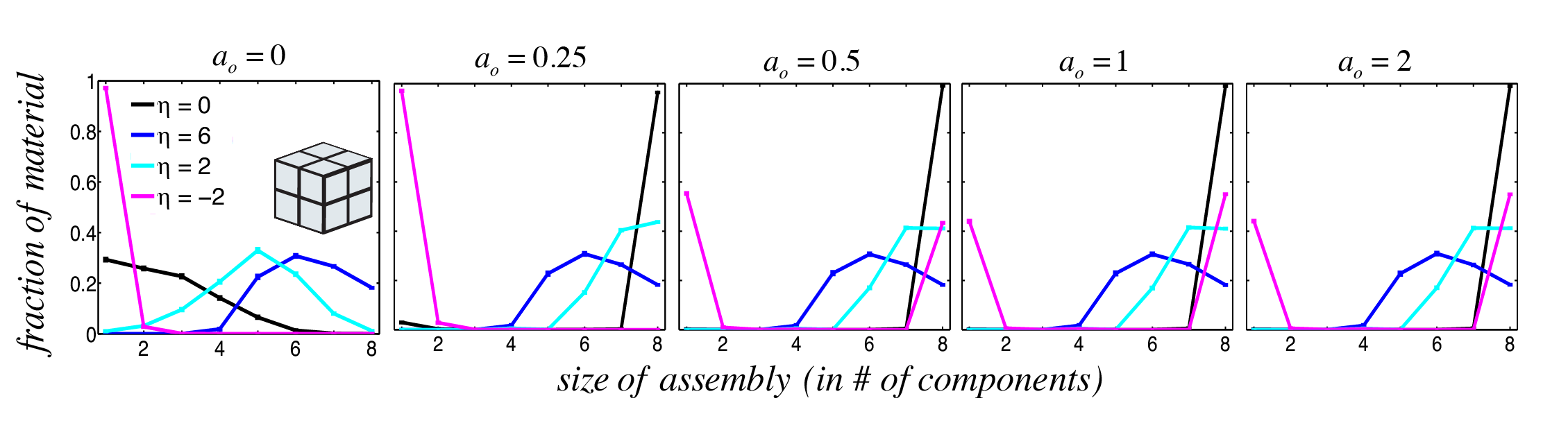

Supplement: Figure S23 — Assembly size distributions for 2×2x2 cube complex at various isothermal conditions and bond coupling constants. All plots are shown after . Inset diagram depicts the complex. (TIF) [file pone.0111233.s023.tif]
