# Supplementary material for: An Assembly Funnel Makes Biomolecular Complex Assembly Efficient
Source: PLoS One. 2014 Oct 31;9(10):e111233. doi: 10.1371/journal.pone.0111233 (PMC4215988; doi:10.1371/journal.pone.0111233)
Supplement: Table S1 — Complex specifics and parameter space explored in this work. (DOCX) [file pone.0111233.s024.docx]

Table S 1 Complex specifics and parameter space explored in this work

| Complex | Number of Components | Number of Valid Species | Number of Reactions | Number of each Starting Component | Iterations |
| --- | --- | --- | --- | --- | --- |
| 1x3 | 3 | 6 | 4 | 10000 | 10 |
| 1x4 | 4 | 10 | 10 | 10000 | 10 |
| 1x5 | 5 | 15 | 20 | 10000 | 10 |
| 1x6 | 6 | 21 | 35 | 10000 | 10 |
| 1x7 | 7 | 28 | 56 | 10000 | 10 |
| 1x8 | 8 | 36 | 84 | 10000 | 10 |
| 1x9 | 9 | 45 | 120 | 10000 | 10 |
| 2x2* | 4 | 13 | 18 | 10000 | 10 |
| 3x3 | 9 | 218 | 1381 | 10000 | 10 |
| 4x4 | 16 | 11506 | 224305 | 10000 | 10 |
| 5x5 | 25 | 2301877 | 115804894 | 1000 | 10 |
| 2x2x2 | 8 | 167 | 1079 | 10000 | 10 |
| Spiral 2x2 | 4 | 14 | 21 | 10000 | 10 |
| Spiral 3x3 | 9 | 281 | 1941 | 10000 | 10 |
| Spiral 4x4 | 16 | 16898 | 357022 | 10000 | 10 |

*This complex is also enumerated in [18] and has an identical list of possible species and reactions to the work presented here
