# Supplementary material for: An Assembly Funnel Makes Biomolecular Complex Assembly Efficient
Source: PLoS One. 2014 Oct 31;9(10):e111233. doi: 10.1371/journal.pone.0111233 (PMC4215988; doi:10.1371/journal.pone.0111233)
Supplement: Table S2 — Criteria for labeling assembly regimes. (DOCX) [file pone.0111233.s025.docx]

Table S 2 Criteria for labeling assembly regimes

| **Color** | **Regime** | **Definition** |
| --- | --- | --- |
|  | Above $T_{m}$ of complex | $y_{eq}<50\%$ |
|  | Nucleation-limited conditions | $y_{eq}\geq50\%,$  $y_{\tau=1000}<0.8y_{eq},$  $\bar{N}_{int, \tau=1000}\leq0.5N_{cplx}$ |
|  | Assembly funnel | $y_{eq}\geq50\%,$  $y_{\tau=1000}\geq0.8y_{eq}$ |
|  | Parallel assembly pathway and rearrangement-limited conditions | $y_{eq}\geq50\%,$  $y_{\tau=1000}<0.8y_{eq},$  $\bar{N}_{int, \tau=1000} >0.5N_{cplx}$ |
