# Supplementary material for: An Assembly Funnel Makes Biomolecular Complex Assembly Efficient
Source: PLoS One. 2014 Oct 31;9(10):e111233. doi: 10.1371/journal.pone.0111233 (PMC4215988; doi:10.1371/journal.pone.0111233)
Supplement: Text S1 — Supporting Methods. (DOCX) [file pone.0111233.s026.docx]

# Text S1 Supporting Methods

## Enumeration Algorithms

We model the self-assembly of 1D “line” complexes, 2D “square grid” complexes, 2D “spiral” complexes, and 3D “cube” complexes. In each of the complexes we model, every component in each complex is distinct. For a given complex, we assume that any connected set of components smaller than the full complex is a valid intermediate and any reaction involving two such species (either components or intermediates) that forms another valid intermediate is allowed. To ensure that all possible assembly and disassembly reactions could occur during our kinetic simulations, we built a full list of what intermediate assemblies could form while assembling a complex and what reactions are allowed. We describe our methods enumerating complex intermediates and assembly reactions below.

## Intermediate Enumeration

To enumerate all valid intermediate assemblies (*i.e.,* species with more than one component) for a given complex, we iterate through all possible subsets of components in the complex. A valid intermediate has full connectivity (*i.e.,* every component in the intermediate shares at least one edge with another component of the subset, see Figure S1). This definition means that intermediates of 2D and 3D complexes may have voids. We chose to include these intermediates because there is experimental evidence that they are present as assembly intermediates [1].

## Reaction Enumeration

After enumerating all valid intermediate assemblies, we determine which species can interact to form larger assembly products. We assume all reactions are reversible and binary (*i.e.*, every forward reaction has two reactants and one product). Valid reactions are those in which two valid species interact given (1) they share no component and (2) the product of the reaction is also a valid species (Figure S2). If both of these criteria are satisfied the reaction is included in the list of possible reactions. We assume aggregation reactions (*i.e.,* those involving two valid intermediate assemblies such as in Figure S2b) are valid because experimental evidence suggests nature often assembles structures via parallel pathways [2-5], which has also been demonstrated as an effective *in vitro* and synthetic assembly strategy [6-8].

[3]

## Kinetic Simulations

The macroscopic forward and reverse reaction rate constants are defined respectively by:

|  | $k_{on}=k_{f},$ | (1) |
| --- | --- | --- |
|  |  |  |
|  | $k_{off,b_{i}}=k_{f}\exp\left( \frac{(\alpha+b_{i})\Delta G^{o}}{RT} \right),$ | (2) |

where $R$ is the universal gas constant, $T$ is absolute temperature, and $\alpha$ is a bond coupling term. Bond coupling is a function of both the number of bonds formed in the$i^{th}$ reaction,$b_{i}$, and a constant parameter, $a_{0}$. Coupling is defined as:

|  | $\alpha\left( b_{i} \right)=\left( a_{o}-1 \right)\left( b_{i}-1 \right),$ | (3) |
| --- | --- | --- |

where $\alpha=0$ for all single bond interactions. For 1D complexes, all reactions happen via a single bond so the value of the constant parameter, $a_{0}$, does not affect the reaction. In 2D and 3D simulations, we vary the parameter $a_{o}$ from ${0\leq a}_{o}\leq2$ in order to model a zero, negative and positive values of bond coupling.

For the reaction of $A+B\leftrightharpoons C$ through $b_{i}$ bonds, the macroscopic reaction rates are:

|  | $r_{on}=k_{on}\left[ A \right]\left[ B \right],$ | (4) |
| --- | --- | --- |
|  | $r_{off,b_{i}}=k_{off,b_{i}}\left[ C \right],$ | (5) |

where $[A]$, [B] and [C] are the respective concentrations of species $A$, $B$ and $C$.

We use stochastic kinetic simulations of the dynamics of self-assembly in which we sample assembly trajectories of the complex. This method enables us to obtain accurate results without requiring tremendous computational resources, as would be the case when integrating many (tens to millions, see Table S1) highly coupled ordinary differential equations of the mass action kinetics model.

For a stochastic kinetic simulation, macroscopic rates must be converted into reaction propensities. The microscopic forward reaction propensity constant is defined as $h_{f}=\frac{k_{f}}{N_{A}V} \frac{1}{\#molecules \cdot second}$ such that the microscopic forward and reverse reaction propensities are defined respectively:

|  | $h_{on}=h_{f}A_{\#}B_{\#},$ | (6) |
| --- | --- | --- |
|  | $h_{off,b_{i}}=h_{f}\exp\left( \frac{\left( \alpha+b_{i} \right)\Delta G^{o}}{RT} \right)C_{\#},$ | (7) |

where $N_{A}$ is Avogadro’s number, $V$ is the volume of the reaction vessel and $A_{\#}$, $B_{\#}$and $C_{\#}$ are number of molecules of A, B and C, respectively. For a given simulation, the initial number of all components is the same. The volume of the reaction vessel is fixed throughout the reaction.

Reaction time in seconds, $t$, is nondimensionalized using parameter $\tau$ such that for the starting component concentration, $\left[ X \right]_{0}$ and the macroscopic forward reaction rate constant, $\tau$ is given by:

|  | $\tau=k_{f}\left[ X \right]_{0}t.$ | (8) |
| --- | --- | --- |

Likewise, reaction temperature is nondimensionalized by the on and off reaction rates through a parameter, $\eta$, where:

|  | $\eta\equiv\log_{10} (\frac{k_{on}}{k_{off,1}}\left[ X \right]_{0}).$ | (9) |
| --- | --- | --- |

High values of $\eta$ correspond to strong interaction strengths (low temperatures) and low values of $\eta$ correspond to weak interaction strengths (high temperatures).

Together, $\eta$ and $\tau$ define the reaction conditions for the self-assembly of a given complex. Specifically, the initial concentration of components, reaction time and temperature together correspond to a particular $\eta$ and $\tau$.

We use Gillespie’s algorithm [9] to sample trajectories of the stochastic kinetics of a given reaction. Because the trajectories are all different, we perform multiple simulations for each complex to obtain a sample of the space of trajectories. Ten simulations are performed for every set of reaction conditions in order to obtain assembly yield average and error bars. Error bars indicate the standard deviation of the reported quantity. For all complexes except the 5x5 square grid, error bars are <1% of the mean.

To report specific assembly times and temperatures, we chose specific reaction rates and concentrations that are inspired by published self-assembly experiments using biomolecular components. We use a diffusion-limited forward reaction rate constant of $k_{f}=6\times{10}^{5}\frac{1}{M sec}$ for all reactions, a value close to those measured for oligonucleotide [10], protein [11], DNA tile [12], and ribosomal subunit-RNA [13] reactions. In practice, the rate constants of reactions involving large assemblies are likely to be slower, reflecting slower diffusion rates. As such, this assumption likely overestimates yields under some conditions.

We chose the standard energetic parameters for two 5 base-pair DNA-DNA “sticky end” hybridization reactions in our definition of $\Delta G^{o}=\Delta H^{o}-T\Delta S^{o}$ where $\Delta H^{o}=-102.4 kcal/mol$, $\Delta S^{o}=-0.3 kcal/mol/K$ which are typical free energy parameters as predicted by the nearest-neighbor model [14]. These values are also similar to a wide variety of protein-ligand complexes [15] and protein-protein interactions in complex formation [16].

In this work, the conformational entropy of the system, similar to conformational entropy in protein folding [17], is a measure of the distribution of occupied energy states in a system and is given by the Boltzmann sampling over all states:

|  | $S=-R\sum_{j} \sum_{i} f_{i,j}\ln\left( f_{i,j} \right)$ | (10) |
| --- | --- | --- |

where $R$ is the universal gas constant and $f_{i,j}$is the fraction of species with energy $i$ and $j$ number of components. Like conformational entropy in protein folding, the conformational entropy in Equation S10 does not take into account other entropic effects, such as molecular vibration within a species. It is therefore not a direct measure of total system entropy.
