# Supplementary material for: An Assembly Funnel Makes Biomolecular Complex Assembly Efficient
Source: PLoS One. 2014 Oct 31;9(10):e111233. doi: 10.1371/journal.pone.0111233 (PMC4215988; doi:10.1371/journal.pone.0111233)
Supplement: Text S2 — Computational Specifics. (DOCX) [file pone.0111233.s027.docx]

# Text S2 Computational Specifics

All enumeration algorithms and stochastic simulations were performed using custom code written in C. Within the C code, multiple optimization techniques were employed in order to decrease computation time. The code used to simulate assembly of the 5x5 square grid complex especially required optimization because more than 30 million possible species can be formed during assembly and roughly 100 million highly coupled reactions are possible (see Table S1). The complexity of this reaction process made the reaction selection step in simple Gillespie algorithm excruciatingly slow. All simulations ran on an in-house server with 8 cores (2 x Quad Core Intel**®** i7 960) and 24 Gb RAM. Thermodynamic equilibrium was calculated using a Gauss-Newton algorithm implemented in MATLAB for smaller complexes (less than 16 components). For larger complexes, we implemented an iterative algorithm in C that converged to thermodynamic equilibrium by choosing a reaction at random (with equal probability to choose any reaction) then zeroing the net reaction rate by changing the species concentrations. The algorithm converged to a final solution when the net reaction rate for all reactions was zero to machine precision (see Text S6 for specifics). All code is available upon request.
