# Supplementary material for: An Assembly Funnel Makes Biomolecular Complex Assembly Efficient
Source: PLoS One. 2014 Oct 31;9(10):e111233. doi: 10.1371/journal.pone.0111233 (PMC4215988; doi:10.1371/journal.pone.0111233)
Supplement: Text S3 — Assembly Regime Criteria. (DOCX) [file pone.0111233.s028.docx]

# Text S3 Assembly Regime Criteria

We demarcate various assembly regimes by using the following values: the yield of the complex at thermodynamic equilibrium, or $y_{eq}$, the yield of the complex after $\tau=1000$, or $y_{\tau=1000}$, and the mean size (in number of components) of the intermediates after $\tau=1000$ and number of components in a complex, or $\bar{N}_{int, \tau=1000}$ and $N_{cplx}$, respectively. Table S2 shows the specific criteria for demarcating assembly regimes.

**Above** $\boldsymbol{T}_{\boldsymbol{m}}$ **of complex**

Molecular engineers can effectively design complexes (usually the target end product of self-assembly) using thermodynamic principles to have a low free energy and thus achieve high yields after very long assembly times ($\tau\to\infty$). So designing complexes that are not stable or subjecting components to assembly conditions that give $y_{eq}<50\%$ is unfavorable from an engineering perspective and hence assigned a red label in our plots.

**Nucleation-limited conditions**

Nucleation-limited conditions thermodynamically favor complex formation ($y_{eq}\geq50\%$) but weak component-component interactions limit yields ($y_{\tau=1000}<0.8y_{eq}$). Under these conditions the mean intermediate size (in number of components) is less than or equal to half of the size of the complex after $\tau=1000$ (or $\bar{N}_{int, \tau=1000}\leq0.5N_{cplx}$, see Figure S15), suggesting that nucleation is the primary growth mechanism of complex assembly.

**Assembly funnel**

The regime where an assembly funnel is present (the assembly funnel regime) is the most favorable regime of assembly, where complexes are highly thermodynamically favored ($y_{eq}\geq50\%$) and complexes form rapidly enough to achieve high-yield in finite times ($y_{\tau=1000}\geq0.8y_{eq}$).

**Parallel assembly pathway and rearrangement-limited conditions**

The parallel assembly pathway and rearrangement-limited regime occurs when complexes are highly thermodynamically favored ($y_{eq}\geq50\%$) but the dynamics of assembly are slow ($y_{\tau=1000}<0.8y_{eq}$). Additionally, under these conditions the mean intermediate size (in number of components) is greater than half of the size of the complex after $\tau=1000$ (or $\bar{N}_{int, \tau=1000}>0.5N_{cplx}$, see Figure S15), suggesting that the rearrangement of components between intermediates is necessary to form complexes.
