# Supplementary material for: An Assembly Funnel Makes Biomolecular Complex Assembly Efficient
Source: PLoS One. 2014 Oct 31;9(10):e111233. doi: 10.1371/journal.pone.0111233 (PMC4215988; doi:10.1371/journal.pone.0111233)
Supplement: Text S4 — Assembly Distribution Selection. (DOCX) [file pone.0111233.s029.docx]

# Text S4 Assembly Distribution Selection

In Figure 2 of the main text, ten species are drawn at random from the reaction mixture. The probability of selecting species $j$, $P_{j}$, for a given sample species is proportional to the mean concentration of $j$, $\bar{C}_{j}$, calculated from the results of ten simulations at a given isothermal assembly condition ($\eta$) after $\tau=1000$ such that:

|  | $P_{j}=\frac{\bar{C_{j}}}{\sum_{i} \bar{C}_{i}},$ | (11) |
| --- | --- | --- |

where $\bar{C}_{i}$ is the mean concentration of species $i$ after the same simulations.
