# Supplementary material for: An Assembly Funnel Makes Biomolecular Complex Assembly Efficient
Source: PLoS One. 2014 Oct 31;9(10):e111233. doi: 10.1371/journal.pone.0111233 (PMC4215988; doi:10.1371/journal.pone.0111233)
Supplement: Text S5 — Further Explanation of Bond Coupling Effect on Yield (from Figure 5). (DOCX) [file pone.0111233.s030.docx]

# Text S5 Further Explanation of Bond Coupling Effect on Yield (from Figure 5)

To determine how energetic changes from multi-bond interactions influences yield, we altered the bond coupling between multiple interfaces on a component. As expected, when the bond coupling constant is very small (*e.g.,*$a_{o}=$0), complexes and intermediates are unstable and thus yield is low under all assembly conditions. But when the bond coupling constant is large ${(a}_{o}\geq1)$, complexes and intermediates are very stable and yield is essentially independent of the bond coupling constant for all assembly conditions above a relatively low threshold. This suggests that large values of bond coupling constant are not required for high-yield self-assembly. However, when ${0<a}_{o}<1$, how bond coupling affects yield depends on the assembly conditions.

**Nucleation-limited conditions**

In the nucleation-limited regime, while thermodynamic analysis would predict that a larger degree of coupling would increase yield (see Figure S7) we found that for a 3x3 square grid complex, some coupling (*e.g.,* $a_{o}\approx0.25$) is important in this regime to stabilize intermediate assemblies. However, higher values of the bond coupling constant do not improve yield. The results suggests that in this regime, where the dynamics of assembly are governed by the nucleation rate, increasing intermediate stability past a point of sufficient stability (the specific value depends on complex size and geometry) does not lead to higher nucleation rates.

**Assembly funnel**

Assembly in the assembly funnel regime achieves yields close to thermodynamic equilibrium for all values of bond coupling. Increasing the bond coupling constant from $a_{o}=0$ increases the stability of intermediates and complexes in which some components are bound to at least three neighbors, resulting in higher yield at thermodynamic equilibrium and after finite times. However, above some small value of the bond coupling constant that is dependent on the complex size and geometry, further increasing the constant beyond this value does not increase the yield, or the size of the assembly funnel. For example, the value of bond coupling constant where this occurs for a 3x3 square grid complex is $a_{o}\approx0.5$, and yield is bounded by $y_{\tau=1000}\approx95\%$. Likewise, the assembly funnel regime for the 3x3 square grid complex includes roughly the same range of $\eta$ values for all bond coupling constants larger than this value.

**Parallel assembly pathway and rearrangement-limited conditions**

In a moderately rearrangement-limited regime ($\eta\approx2$), small values of the bond coupling constant can actually improve assembly dynamics by enhancing rearrangement rates (*e.g.,* $a_{o}\approx0.25$ for a 3x3 square grid complex). Here, intermediates are less stable, which results in increased rates of disassembly and hence higher yields than assembly under the same conditions but with a higher bond coupling constant. While enhanced yield is possible under moderately rearrangement-limited regimes, under effectively irreversible conditions ($\eta\approx6$) yield is independent of bond coupling because single component-component interactions are so strong that all disassembly is unlikely.
