# Supplementary material for: An Assembly Funnel Makes Biomolecular Complex Assembly Efficient
Source: PLoS One. 2014 Oct 31;9(10):e111233. doi: 10.1371/journal.pone.0111233 (PMC4215988; doi:10.1371/journal.pone.0111233)
Supplement: Text S6 — Computing Thermodynamic Equilibrium of Large Complexes. (DOCX) [file pone.0111233.s031.docx]

# Text S6 Computing Thermodynamic Equilibrium of Large Complexes

This section describes the algorithm used to compute the concentrations of components, intermediates and complexes at equilibrium for larger (16 and 25 component) complexes. We start with an initial guess of component and complex concentrations that preserves mass balance among reaction components with respect to the initial conditions of the reaction. The algorithm then chooses a reaction at random (with equal probability to choose any reaction in the list of possible reactions), and zeros the net reaction rate by computing the change in the species concentrations that would give the concentration of species at equilibrium.

For a given reaction $A+B\leftrightharpoons C$, the equilibrium constant, $K_{eq}$, is given by:

|  | $K_{eq}=\frac{k_{on}}{k_{off,b_{i}}}=\frac{\left[ C \right]_{eq}}{\left[ A \right]_{eq}\left[ B \right]_{eq}}.$ | (12) |
| --- | --- | --- |

The equilibrium concentrations of the species will be $\left[ A \right]_{eq}=\left[ A \right]-\delta$, $\left[ B \right]_{eq}=\left[ B \right]-\delta$ and $\left[ C \right]_{eq}=\left[ C \right]+\delta$, where $\delta$ is some change in concentration and, $\left[ A \right], \left[ B \right]$ and $[C]$ are current species concentrations. One can solve for $\delta$ using the quadratic formula. We update the species concentrations if $\delta$ is both larger than a small threshold value and as a fraction of current species concentration$\delta$ is larger than a small tolerance value. If neither is the case, we deem the reaction to be at equilibrium.

This process of randomly selecting reactions and determining whether the species have achieved their equilibrium concentration continues until enough consecutive sampled reactions (roughly $n^{2}$ for $n$ reactions) are within the tolerance value of the equilibrium point. This ensures that all reactions are equilibrated with high probability. At this point the algorithm terminates.
