# Supplementary material for: An Assembly Funnel Makes Biomolecular Complex Assembly Efficient
Source: PLoS One. 2014 Oct 31;9(10):e111233. doi: 10.1371/journal.pone.0111233 (PMC4215988; doi:10.1371/journal.pone.0111233)
Supplement: Text S7 — Supporting References. (DOCX) [file pone.0111233.s032.docx]

# Supporting References

1. Rothemund PWK (2006) Folding DNA to create nanoscale shapes and patterns. Nature 440: 297-302.

2. Mulder AM, Yoshioka C, Beck AH, Bunner AE, Milligan RA, et al. (2010) Visualizing Ribosome Biogenesis: Parallel Assembly Pathways for the 30S Subunit. Science 330: 673-677.

3. Korevaar PA, George SJ, Markvoort AJ, Smulders MMJ, Hilbers PAJ, et al. (2012) Pathway complexity in supramolecular polymerization. Nature 481: 492-496.

4. Powers ET, Powers DL (2008) Mechanisms of protein fibril formation: Nucleated polymerization with competing off-pathway aggregation. Biophysical Journal 94: 379-391.

5. Pan J, Thirumalai D, Woodson SA (1997) Folding of RNA involves parallel pathways. Journal of Molecular Biology 273: 7-13.

6. Knowles TPJ, Oppenheim TW, Buell AK, Chirgadze DY, Welland ME (2010) Nanostructured films from hierarchical self-assembly of amyloidogenic proteins. Nature Nanotechnology 5: 204-207.

7. Groschel AH, Schacher FH, Schmalz H, Borisov OV, Zhulina EB, et al. (2012) Precise hierarchical self-assembly of multicompartment micelles. Nature Communications 3.

8. Gandra N, Abbas A, Tian LM, Singamaneni S (2012) Plasmonic Planet-Satellite Analogues: Hierarchical Self-Assembly of Gold Nanostructures. Nano Letters 12: 2645-2651.

9. Gillespie DT (1977) Exact Stochastic Simulation of Coupled Chemical-Reactions. Journal of Physical Chemistry 81: 2340-2361.

10. Wetmur JG (1991) DNA Probes - Applications of the Principles of Nucleic-Acid Hybridization. Critical Reviews in Biochemistry and Molecular Biology 26: 227-259.

11. Camacho CJ, Kimura SR, DeLisi C, Vajda S (2000) Kinetics of desolvation-mediated protein-protein binding. Biophysical Journal 78: 1094-1105.

12. Evans CG, Hariadi RF, Winfree E (2012) Direct Atomic Force Microscopy Observation of DNA Tile Crystal Growth at the Single-Molecule Level. Journal of the American Chemical Society 134: 10485-10492.

13. Recht MI, Williamson JR (2001) Central domain assembly: Thermodynamics and kinetics of S6 and S18 binding to an S15-RNA complex. Journal of Molecular Biology 313: 35-48.

14. SantaLucia J (1998) A unified view of polymer, dumbbell, and oligonucleotide DNA nearest-neighbor thermodynamics. Proceedings of the National Academy of Sciences of the United States of America 95: 1460-1465.

15. Cozzini P, Fornabaio M, Marabotti A, Abraham DJ, Kellogg GE, et al. (2002) Simple, intuitive calculations of free energy of binding for protein-ligand complexes. 1. Models without explicit constrained water. J Med Chem 45: 2469-2483.

16. Horton N, Lewis M (1992) Calculation of the free energy of association for protein complexes. Protein Sci 1: 169-181.

17. Pickett SD, Sternberg MJE (1993) Empirical Scale of Side-Chain Conformational Entropy in Protein-Folding. Journal of Molecular Biology 231: 825-839.

18. Bray D, Lay S (1997) Computer-based analysis of the binding steps in protein complex formation. Proceedings of the National Academy of Sciences of the United States of America 94: 13493-13498.
